# New Environment, New Invaders—Repeated Horizontal Transfer of LINEs to Sea Snakes

**DOI:** 10.1093/gbe/evaa208

**Published:** 2020-10-06

**Authors:** James D. Galbraith, Alastair J. Ludington, Alexander Suh, Kate L. Sanders, David L. Adelson

**Affiliations:** 1 School of Biological Sciences, University of Adelaide, Australia; 2 Department of Ecology and Genetics—Evolutionary Biology, Evolutionary Biology Centre, Uppsala University, Sweden; 3 Department of Organismal Biology—Systematic Biology, Evolutionary Biology Centre, Uppsala University, Sweden; 4 School of Biological Sciences, University of East Anglia, Norwich, United Kingdom

**Keywords:** horizontal transfer, transposable element, Serpentes

## Abstract

Although numerous studies have found horizontal transposon transfer (HTT) to be widespread across metazoans, few have focused on HTT in marine ecosystems. To investigate potential recent HTTs into marine species, we searched for novel repetitive elements in sea snakes, a group of elapids which transitioned to a marine habitat at most 18 Ma. Our analysis uncovered repeated HTTs into sea snakes following their marine transition. The seven subfamilies of horizontally transferred LINE retrotransposons we identified in the olive sea snake (*Aipysurus laevis*) are transcribed, and hence are likely still active and expanding across the genome. A search of 600 metazoan genomes found all seven were absent from other amniotes, including terrestrial elapids, with the most similar LINEs present in fish and marine invertebrates. The one exception was a similar LINE found in sea kraits, a lineage of amphibious elapids which independently transitioned to a marine environment 25 Ma. Our finding of repeated horizontal transfer events into marine snakes greatly expands past findings that the marine environment promotes the transfer of transposons. Transposons are drivers of evolution as sources of genomic sequence and hence genomic novelty. We identified 13 candidate genes for HTT-induced adaptive change based on internal or neighboring HTT LINE insertions. One of these, ADCY4, is of particular interest as a part of the KEGG adaptation pathway “Circadian Entrainment.” This provides evidence of the ecological interactions between species influencing evolution of metazoans not only through specific selection pressures, but also by contributing novel genomic material.

**Significance**

Recent research has found horizontal transfer (HT) of transposons between marine animals. We analyzed the olive sea snake (*Aipysurus laevis*) genome, uncovering HT of six novel retrotransposons into sea snakes since their marine transition within the last 18 Ma. All six are absent from terrestrial animals and are most similar to retrotransposons found in fish, corals, and the independently marine sea kraits. All six retrotransposons are likely still active and expanding across the genome in *A. laevis*. Our findings suggest the marine environment is ideal for the HT of transposons; and provide evidence that changing environments can influence evolution not only through novel selective pressures, but also by contributing novel genomic material.

## Introduction

Transposons are a major component of metazoan genomes, making up between 24% and 56% of squamate genomes (Pasquesi et al. 2018). Transposons are split into two classes: Class I containing LINEs (long interspersed elements) and LTR (long terminal repeat) retrotransposons; and Class II containing DNA transposons (Wicker et al. 2007). Although all three groups of transposons are present in squamates, recent activity is dominated by LINEs including CR1s, RTE-BovBs, Rex1, and L2s (Pasquesi et al. 2018). Although transposons are normally vertically transmitted (parent to offspring) there have been many instances of horizontal transposon transfer (HTT) observed between distantly related species. HTT of DNA transposons and LTR retrotransposons appears to be more common, yet many examples of HTT of non-LTR retrotransposons (LINEs) have been described (Peccoud et al. 2018). These include transfers of RTE-BovBs between multiple distant lineages (Ivancevic et al. 2018), of AviRTEs between birds and parasitic nematodes (Suh et al. 2016), and of Rex1 elements between teleost fish (Volff et al. 2000; Zhang et al. 2020). As transposons proliferate throughout a genome they can contribute novel coding sequences, alter gene regulatory networks, modify coding regions, and lead to gene copy number variation (Rebollo et al. 2012; Chuong et al. 2017; Cerbin and Jiang 2018; Schrader and Schmitz 2019). Within a lifetime most insertions will be neutral and some may be deleterious; however, on an evolutionary time scale, some TE insertions constitute a key source of genomic innovation as organisms adapt to new and changing environments (Casacuberta and González 2013; Salces-Ortiz et al. 2020). Previous studies in *Drosophila* found HTT to increase following colonization of new habitats due to exposure to new species (Biémont et al. 1999; Vieira et al. 2002).

Hydrophiinae (Elapidae) is a prolific radiation of more than 100 terrestrial snakes plus ∼70 aquatic species. The aquatic species form two separate lineages which independently transitioned to a marine habitat: the fully marine sea snakes and the amphibious sea kraits (*Laticauda*) (Lee et al. 2016). Sea snakes are phylogenetically nested inside the terrestrial hydrophiine radiation and appeared ∼6–18 Ma, whereas sea kraits form the sister lineage to all other Hydrophiinae and diverged 25 Ma (Sanders et al. 2008; Lee et al. 2016). Sea snakes include >60 species in two major clades, *Hydrophis* and *Aipysurus-Emydocephalus*, which shared a semi-aquatic common ancestor ∼6–18 Ma and exhibit highly contrasting evolutionary histories since their transitions from terrestrial to marine habits (Sanders et al. 2013; Lee et al. 2016; Nitschke et al. 2018). Both of these lineages have independently developed adaptations to the aquatic environment including a lingual notch allowing for full closure of the mouth when underwater, and tail paddles for efficient underwater movement (Lillywhite 2014). However, the *Aipysurus-Emydocephalus* lineage has continued to evolve at the same rate as terrestrial lineages of Hydrophiinae, diverging into nine species, whereas the *Hydrophis* lineage has rapidly radiated into 48 species (Sanders et al. 2010).

Following major ecological transitions, such as sea snakes’ transition from a terrestrial to a marine habitat, organisms must adapt to their new environment, with transposons potentially being a key genomic source for genomic adaptations (Schlötterer et al. 2015; Marques et al. 2018). Peng et al. (2020) found expansions of LTR retrotransposons in Shaw’s sea snake (*Hydrophis curtus*) to be linked to its adaptation to the marine environment. This was based on overrepresentation of GO terms of genes near inserted LTR retrotransposons and found potential links to locomotory behavior, eye pigmentation, cellular hypotonic response, positive regulation of wound healing, and olfactory bulb interneuron development. Here we analyzed transposons in three sea snake genomes and one sea krait genome, where the marine environment appears to have fostered the repeated, independent acquisition of these transposons through HTT. The repeated HTT suggests that direct effects of the environment on genome structure may be an important but overlooked driver of evolutionary change during major ecological transitions.

## Results

### Annotation of Sea Snake Transposons

We performed ab initio repeat annotation of the olive sea snake (*Aipysurus laevis*) genome (Ludington et al., dx.doi.org/10.5281/zenodo.3975254) using CARP (Zeng et al. 2018) and RepeatModeler (Smit and Hubley 2017) to characterize repetitive content. Most repetitive sequences identified by both CARP and RepeatModeler were not well classified because both software tools rely on homology to reference sequences from Repbase (Bao et al. 2015), a database of repeats from highly studied species that are evolutionarily distant to Hydrophiinae. The reliance on sequence homology alone for genome-wide repeat annotation of newly sequenced species often results in the incorrect and misannotation of repeats (Platt et al. 2016). We used a structural homology approach based on the presence of a variety of protein domains in these poorly annotated repeats to identify subfamilies of LINEs, Penelope and LTR retrotransposons, endogenous retroviruses, and DNA transposons. Consensus sequences containing the characteristic protein domains and, if appropriate, TIRs or LTRs were considered as full length and confidently assigned to the lowest Transposable Element (TE) taxonomy level possible. For example, sequences identified as containing 90% of a reverse transcriptase domain and 90% of an endonuclease domain were classified as LINEs.

**Fig. 1.**
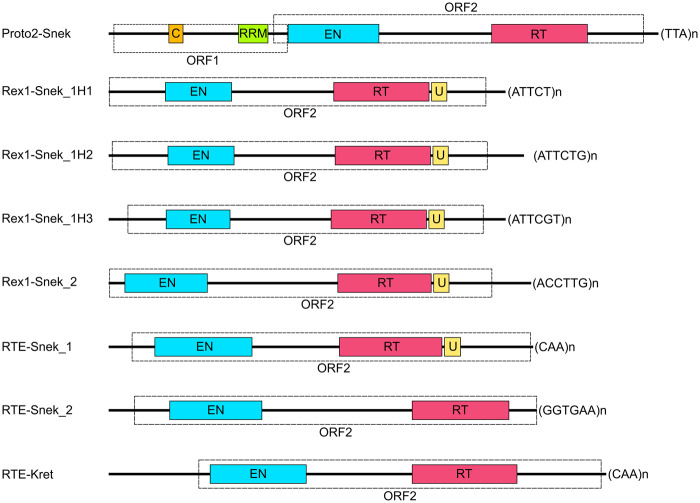
Structure of the seven HTT *Aipysurus* and one *Laticauda* LINE subfamilies. Cyan represents endonuclease (EN), red reverse transcriptase (RT), orange coiled coil (CC), green RNA-recognition motif (RRM), and yellow domain of unknown function 1891 (U). Protein domains were identified using RPSBLAST (Marchler-Bauer et al. 2017) and HHpred (Zimmermann et al. 2018) searches against CDD and Pfam (Finn et al. 2016; Marchler-Bauer et al. 2017) databases and the coiled-coil domain was identified using PCOILS (Gruber et al. 2006).

To identify potential HTT events which may have occurred since the transition of elapids to a marine habitat, we looked for transposons identified in *A. laevis* that were not present in genome assemblies of its closest sequenced terrestrial relatives, *Notechis scutatus* (tiger snake) and *Pseudonaja textilis* (eastern brown snake). All the TE subfamilies characterized in the *A. laevis* genome were found to be present in *P. textilis* and *N. scutatus* with the exception of five LINE subfamilies discussed below (see fig. 1). These subfamilies were further classified based on CENSOR (Kohany et al. 2006) searches against Repbase (Bao et al. 2015) using the online interface. Consensus sequences containing the characteristic protein domains were confidently assigned to the lowest TE taxonomy level possible.

**Fig. 2.**
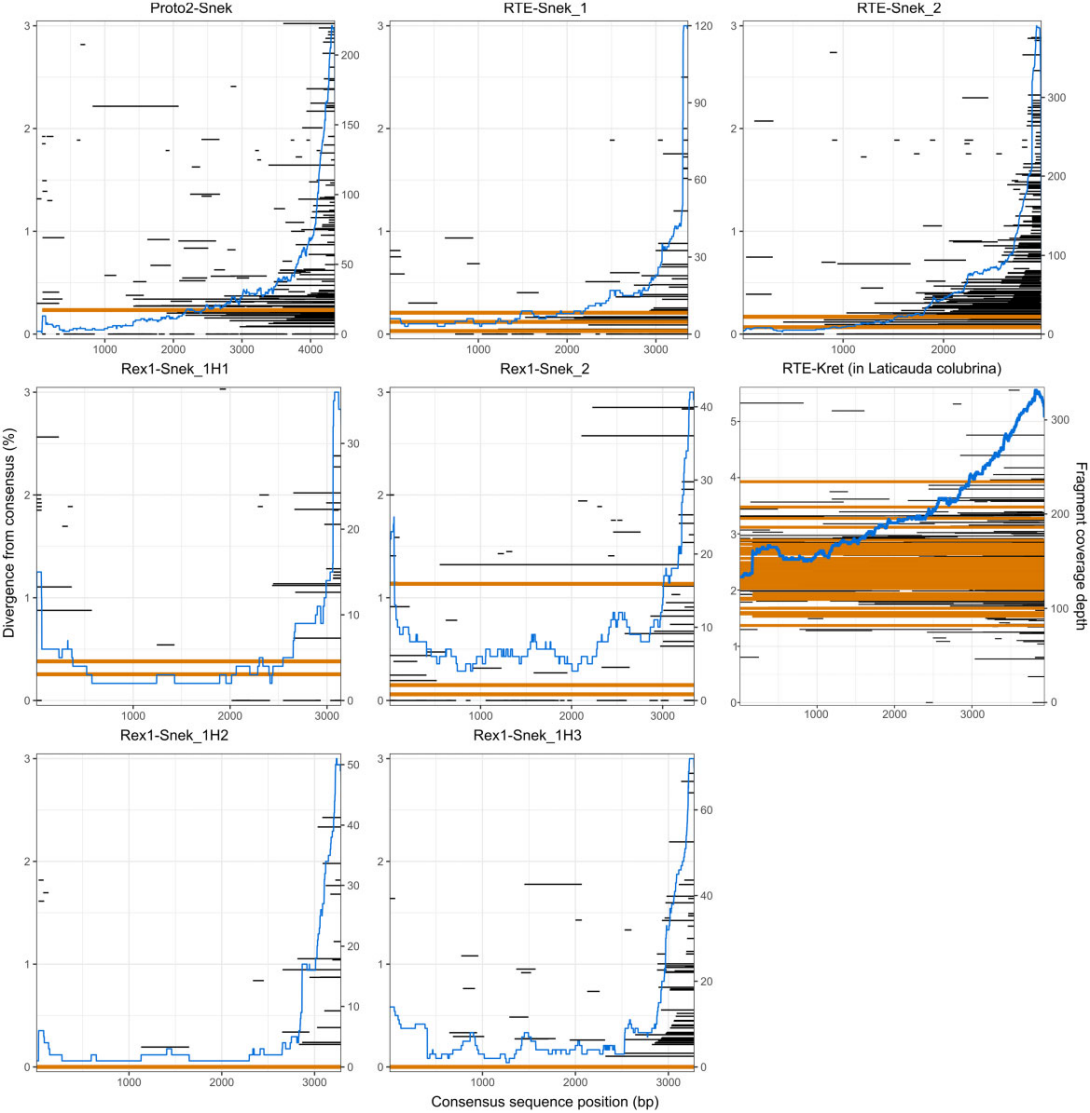
Coverage and divergence from consensus of the seven horizontally transferred LINE subfamilies identified in the *Aipysurus laevis* genome and the one identified in *Laticauda colubrina*. LINE fragments were identified with BLASTN (Altschul et al. 1990; Camacho et al. 2009) and plotted using ggplot2 (Wickham 2011) using the consensus2genome script (https://github.com/clemgoub/consensus2genome, last accessed September 16, 2020). The blue line represents the depth of coverage of fragments aligned to the subfamily consensus sequence (shown on right-hand *y* axis). Each horizontal line represents the divergence of a fragment and its position mapped to the repeat consensus (position shown on *x* axis); orange shows full-length repeats and black shows repeat fragments. The divergence from consensus of the repeats is shown on the left-hand *y* axis.

In *A. laevis* two of the five LINEs subfamilies, Rex1-Snek_1 (five full-length copies found) and Rex1-Snek_2 (three full-length copies found) belong to the CR1/Jockey superfamily but share less than 100-bp nucleotide sequence homology. Manual curation (see Methods) of a multiple sequence alignment of the five full-length copies identified by CARP revealed Rex1-Snek_1 to be three subfamilies; henceforth named Rex1-Snek_1H1, Rex1-Snek_1H2 and Rex1-Snek_1H3. Rex1-Snek_1H2 and Rex1-Snek_1H3 have 90% and 89% pairwise identity with Rex1-Snek_1H1, respectively. The other three subfamilies, RTE-Snek_1 (three full-length sequences found), RTE-Snek_2 (one full-length sequence found), and Proto2-Snek (one full-length sequence found) belong to the RTE superfamily but have no significant nucleotide sequence homology based on BLASTN searches using default parameters. In addition to the full-length sequences, we identified hundreds of highly similar copies with 5ʹ truncation patterns characteristic of recently active LINEs (fig. 2, supplementary tables 1 and 2, Supplementary material online). Specifically, coverage plots of the RTE-Snek_1, RTE-Snek_2, and Proto2-Snek families are typical of LINEs, with a clear pattern of 5ʹ-truncated insertions (Luan et al. 1993). All seven LINE subfamilies were most similar to Repbase TE reference sequences from a marine annelid worm, a marine crustacean, and teleost fishes (Bao et al. 2015) (see table 1, supplementary dataset 1, Supplementary material online).

**Table 1:** Most Similar Repbase and Curated Repeats for Each LINE Subfamily in Species Outside of Closely Related Snakes

Repeat (query)	Species (target repeat)	Percent identity	Hit length (bp)
Most similar Repbase sequences			
Rex-Snek_1H1	*Petromyzon marinus* (Rex1-1_PM)	67.5	1,359
Rex-Snek_1H2	*Petromyzon marinus* (Rex1-1_PM)	66.7	1,359
Rex-Snek_1H3	*Petromyzon marinus* (Rex1-1_PM)	64.2	2,796
Rex-Snek_2	*Cyprinus carpio* (Rex1-1_CCa)	75.9	2,795
RTE-Snek_1	*Petromyzon marinus* (RTE-2_PM)	62.9	3,100
RTE-Snek_2	*Chrysemys picta* (RTE-9_CPB)	65.3	2,926
Proto2-Snek	*Oryzias latipes* (Proto2-1_OL)	65.6	666
RTE-Kret	*Petromyzon marinus* (RTE-2_PM)	63.6	3,102
Most similar curated repeats			
Rex-Snek_1H1	*Oryzias latipes*	85.0	2,987
Rex-Snek_1H2	*Oryzias latipes*	82.2	2,973
Rex-Snek_1H3	*Oryzias latipes*	81.6	2,960
Rex-Snek_2	*Miichthys miiuy*	78.7	2,594
RTE-Snek_1	*Laticauda colubrina* (RTE-Kret)	84.9	3,252
RTE-Snek_2	*Hippocampus comes*	74.4	3,184
Proto2-Snek	*Epinephelus lanceolatus*	75.4	3,299
RTE-Kret	*Aipysurus laevis* (RTE-Snek_1)	84.9	3.252

Note.—Repbase was searched using the seven consensus *Aipysurus laevis* LINEs using relaxed BLASTN parameters (see Materials and Methods). A database of our curated repeats from all searched species (see Materials and Methods) was searched using the seven consensus *A. laevis* repeats using default BLASTN parameters.

**Table 2:** HTT LINEs Inserted into Exons, UTRs, or within 5,000 bp Upstream of 5ʹ UTRs of Genes within the *A. laevis* Assembly and Transcriptome

Gene	LINE	Distance to 5’ UTR (bp)	Insertion size (bp)
Acetyl-CoA Acyltransferase 1 (*ARIH1*)	Proto2-Snek	223	85
KN Motif And Ankyrin Repeat Domains 4 (*KANK4*)	Proto2-Snek	4,987	161
Potassium Calcium-Activated Channel Subfamily N Member 4 (*KCNN4*)	Proto2-Snek	3,746	98
Outer Mitochondrial Membrane Lipid Metabolism Regulator OPA3 (*OPA3*)	Rex1-Snek_2	3,149	81
Rabaptin, RAB GTPase Binding Effector Protein 1 (*RABEP1*)	Proto2-Snek	1,389	99
Valosin Containing Protein Lysine Methyltransferase (*VCPKMT*)	Rex1-Snek_1H1	512	76
Cdc42 effector protein 4 (*CDC42EP4*)	RTE-Snek_2	1,475	422
Gamma-aminobutyric acid receptor subunit alpha-3 (*GABRA3*)	RTE-Snek_2	4,247	95
Leucine-zipper-like transcriptional regulator 1 (*LZTR1*)	RTE-Snek_2	2,066	421
Polyadenylate-binding protein 2 (*PABPN1*)	RTE-Snek_2	145	431
Parvalbumin alpha (*PVALB*)	RTE-Snek_2	4,152	52
Deaminated glutathione amidase (*NIT1*)	RTE-Snek_2	In coding exon	228
Adenylate cyclase type 4 (*ADCY4*)	RTE-Snek_2	In 3ʹ UTR and transcript	130
CAP-Gly Domain Containing Linker Protein Family Member 4 (*CLIP4*)	RTE-Snek_1	In transcript	—
BLOC-1 Related Complex Subunit 8 (*BORCS8*)	Rex1-Snek_1H3	In transcript	—

The absence of these recently active LINE subfamilies from terrestrial snakes that shared a common ancestor with sea snakes within the last approximately 18 Ma, combined with the finding that they were most similar to LINEs from distantly related aquatic organisms, suggested HTT as the most plausible explanation. There are three diagnostic features of HTT: 1) the sporadic presence of a TE family within a set of closely related species, 2) a higher than expected degree of sequence identity in long diverged species, and 3) discordant topologies for the phylogenies of transposons and their host species (Silva et al. 2004).

### Presence/Absence in Closely Related Species

**Fig. 3.**
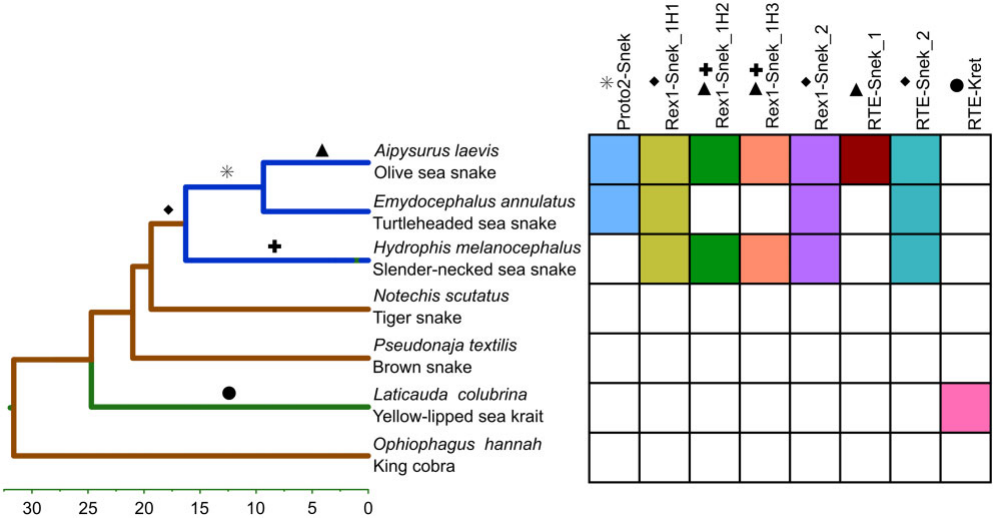
Presence of the eight HTT LINE subfamilies across the phylogeny of elapid snakes (adapted from Lee et al. [2016]). Color of lineage represents habitat—marine species are blue, terrestrial brown, and amphibious green. Each symbol represents the likely timing of horizontal transfers, for example, the square indicates the likely transfer date of both Rex1-Snek_1H1 and Rex1-Snek_2. Presence/absence determined using reciprocal BLASTN search (Altschul et al. 1990; Camacho et al. 2009) using default parameters.

As mentioned above, the seven LINE subfamilies were absent from the closest terrestrial relatives of *A. laevis*. To test if the subfamilies have a sporadic distribution in closer relatives, we performed reciprocal BLASTN searches for their presence in two closely related sea snake genome assemblies, *Hydrophis melanocephalus* (black-headed sea snake) and *Emydocephalus ijimae* (Ijima's turtle-headed sea snake); the two closest (available) terrestrial species, *N. scutatus* and *P. textilis*; an independently aquatic species, *Laticauda colubrina* (yellow-lipped sea krait); and a distant terrestrial relative, *Ophiophagus hannah* (king cobra). The reciprocal search for RTE-Snek_1 revealed a similar yet distinct RTE subfamily present in *L. colubrina*, henceforth referred to as RTE-Kret. From these searches, we found RTE-Snek_1 was restricted to *A. laevis* and RTE-Kret to be restricted to *L. colubrina*. In addition to being present in *A. laevis*, Proto2-Snek was also present in *E. ijimae*; Rex1-Snek_1H1, Rex1-Snek_2, and RTE-Snek_2 in *E. ijimae* and *H. melanocephalus*; and Rex1-SnekH2 and Rex1-SnekH3 in *H. melanocephalus*. This reciprocal search confirmed all seven subfamilies were absent from both terrestrial (*N. scutatus*, *P. textilis*, and *O. hannah*) and aquatic (*L. colubrina*) outgroups, and RTE-Kret was restricted to *L. colubrina* (fig. 3, supplementary figs. 1–8, Supplementary material online).

**Fig. 4.**
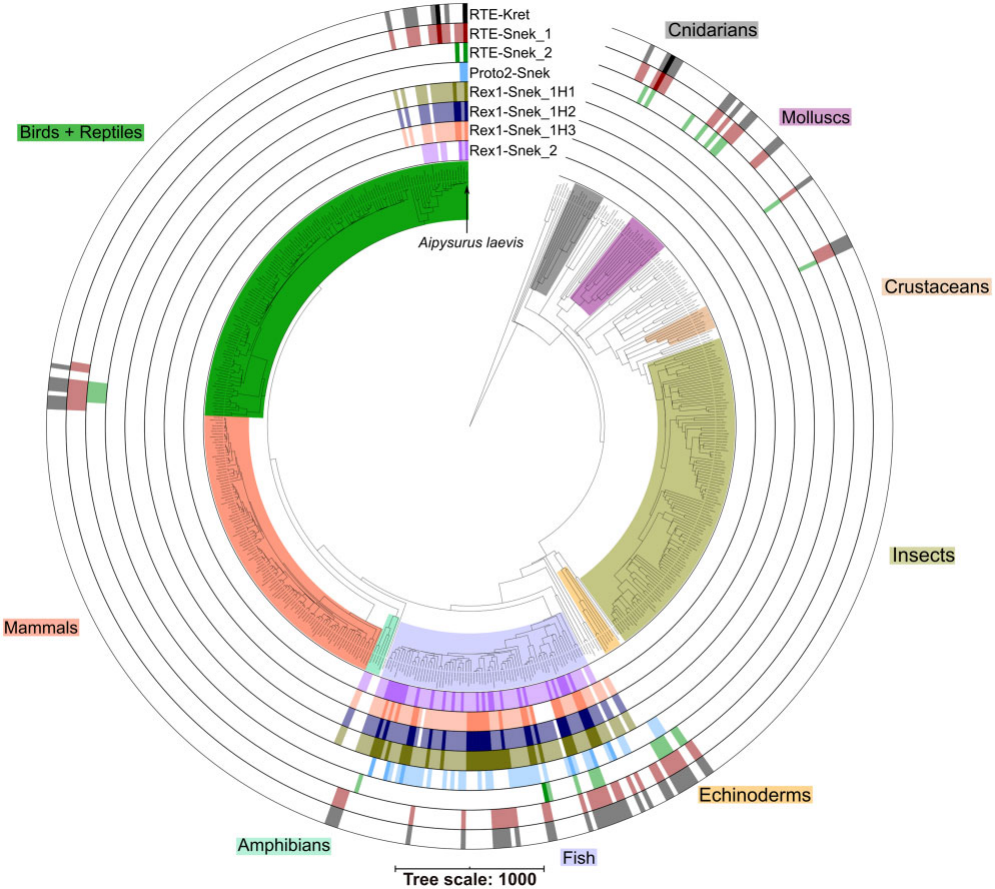
Presence of the seven *Aipysurus* and one *Laticauda* HTT LINE subfamilies across 540 Metazoa. In each ring, darker shading represents the presence of at least one sequence over 1,000 bp in length showing 75% or higher pairwise identity to the LINE, lighter shading represents the presence of more than one sequence over 1,000 bp with less than 75% pairwise identity, and white represents the complete absence of similar sequences. Presence of LINEs identified using BLASTN with custom parameters (see Materials and Methods) (Altschul et al. 1990; Camacho et al. 2009) and plotted in iToL (Letunic and Bork 2019). Species tree generated using TimeTree (Hedges et al. 2006), manually edited to correct elapid phylogeny to fit (Lee et al. 2016). Interactive tree available at https://itol.embl.de/shared/jamesdgalbraith (last accessed September 16, 2020).

**Fig. 5.**
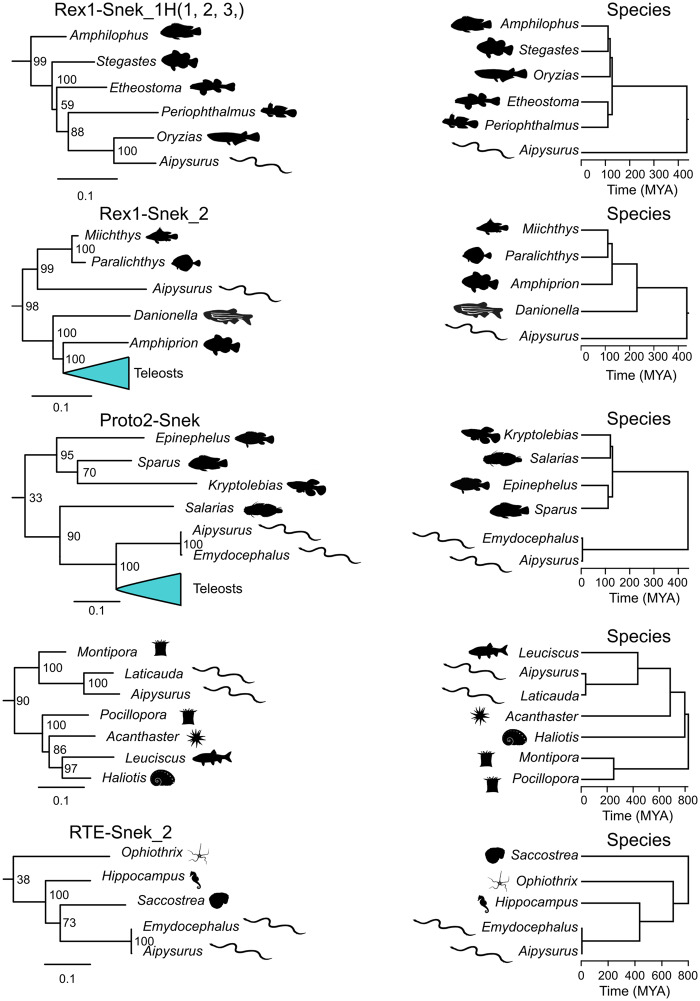
Excerpts from the phylogenies of all intact curated and Repbase RTEs and all intact curated and Repbase Rex1s compared with host species phylogeny. The blue triangles on the left represent condensed large subtrees of LINE sequences. TE phylogeny scale bar represents substitutions per site. The numbers next to each node in the repeat trees are the support value. Extracts from larger phylogenies constructed using RAxML (Stamatakis 2014) based on MAFFT (Katoh and Standley 2013) nucleotide alignments trimmed with Gblocks (Talavera and Castresana 2007) (for full phylogenies see supplementary Appendix, figs. S1 and S2, Supplementary material online). Species trees constructed with TimeTree (Hedges et al. 2006).

We used two approaches to estimate the number and timing of HTT events into sea snakes. Based on the presence or absence of the seven *A. laevis* LINEs in *O. hannah*, *L. colubrina*, *P. textilis*, *N. scutatus*, *H. melanocephalus*, *E. ijimae*, and *A. laevis*, we conservatively estimated nine HTT events into sea snakes dated using the species divergence times from Sanders et al. (2008, 2009, 2013) and Lee et al. (2016) (fig. 3, supplementary table 2, Supplementary material online). Due to the lack of fragments of Rex1-Snek_1H2 and Rex1-Snek_1H3 in *Emydocephalus* (supplementary figs. 10 and 11, Supplementary material online), these two subfamilies were likely transferred independently into *Aipysurus* and *Hydrophis*. In addition, we calculated the timing of HTT into the *Aipysurus* lineage using the average substitutions per site of each LINE subfamily and an estimated genome-wide substitution rate. The insertion time based on substitution rate (supplementary table 2, Supplementary material online) suggests that the HTTs postdate the divergence of *Aipysurus* and *Emydocephalus*. Taking the high standard deviation into account, the timing of HTT events estimated by both methods overlapped with the exception of the transfer of RTE-Snek_2 (supplementary table S2, Supplementary material online).

As an independent verification of presence/absence and to look for potential current activity of the LINEs, we searched assembled transcriptomes of a variety of tissues from three sea snakes—*A. laevis*, *A. tenuis*, and *Hydrophis major* from Crowe-Riddell (2019) (see supplementary dataset 2, Supplementary material online). We identified high-identity transcripts (>95% identity) of all Rex1-Snek1H1, Rex1-Snek1H2, Rex1-Snek1H3, Rex1-Snek_2, and RTE-Snek_2 in at least one tissue of *A. laevis*, *A. tenuis*, and *H. major*. High-identity transcripts of RTE-Snek_1 and Proto2-Snek were present in *A. laevis* and *A. tenuis*, yet absent from all *H. major* tissues, with one small fragment of an RTE-Snek_1-like transcript present in an *H. major* testis transcriptome. The presence of transcripts of all seven LINE subfamilies both confirmed the presence/absence pattern of the specific subfamilies in *A. laevis* and indicates potential ongoing retrotransposition of these elements.

### Verification of HTT and Search for HTT Donor Species

Although the absence of the marine-specific TEs in close terrestrial species supported HTT to sea snakes, we needed to rule out the possibility that those TEs were lost from those terrestrial species. In order to confirm HTT versus loss of TEs, we searched for all seven LINE subfamilies in 630 metazoan genomes using BLASTN with relaxed parameters (see Materials and Methods). Our search identified homologous, yet divergent Rex1s in fish and squamates, Proto2s in fish, and RTEs widespread across a variety of marine organisms including fish, echinoderms, corals, and sea kraits (see fig. 4, supplementary dataset 4, Supplementary material online). Using these hits as seeds, we curated consensus repeats of each LINE subfamily in the species they were identified in.

We then aligned our original LINE sequences against a database containing both our curated repeats and Repbase repeats. All seven of our original LINE subfamilies were most similar to curated LINEs found in marine species (table 1) with pairwise identity for all closest hits between 75–85%. Rex1-Snek_1H1, Rex1-SnekH2, Rex1-SnekH3, and Rex1-Snek_2 were most similar to Rex1s curated from a variety of fish genomes. Proto2-Snek was most similar to a Proto2 from the European carp (*Cyprinus carpio*) genome and RTE-Snek_1 most similar to RTE-Kret from *L. colubrina*. If the LINE subfamilies were present in sea snakes yet absent from terrestrial and amphibious elapids due to repeated losses, we would expect to find highly similar LINEs to still be present in other squamates. However, we failed to identify highly similar repeats in any squamates; therefore, the most parsimonious explanation supports HTT and rules out loss. We used the results of this search in an attempt to identify the likely donor or vector species by looking for species hosting our HTT LINEs with a comparable degree of sequence divergence to that observed in *A. laevis*. However, none of the cross-species alignments were greater than 87% nucleotide sequence identity and therefore did not show comparable sequence divergence which would be required to identify potential donor species (table 1).

### Discordant Phylogenies of RTEs and of Rex1s Compared with Host Species

As extreme discordance between repeat and species phylogenies would further support HTT, we compared the respective tree topology of all RTEs, Proto2s, and Rex1s, using both Repbase sequences and our curated sequences, to the species tree topology. As illustrated in figure 5, the species and repeat phylogenies of all seven sea snake LINE subfamilies and the *L. colubrina* RTE are highly discordant, evidenced by their clustering with teleost fishes. This confirms likely HTT events from marine organisms into sea snakes and sea kraits, and further refutes independent losses from terrestrial Australian elapids.

### Insertions in and Near Coding Regions

To identify any insertions of these LINEs in *A. laevis* which may have the potential to alter gene expression or protein structure, we identified all insertions in or near regions annotated as genes, in particular exons and untranslated regions (UTRs) (supplementary table 1, Supplementary material online). Intersects of gene and repeat annotation intervals in the *A. laevis* assembly initially revealed 23 insertions of HTT LINEs in or near genes: 19 insertions in 5ʹ UTRs or within 5,000 bp upstream, 1 into a coding exon and 3 into 3ʹ UTRs.

To test for potential assembly errors that might have yielded erroneous insertions near genes, we searched for the flanking regions of the 23 insertions in the closely related *E. ijimae* and *H. melanocephalus*. Eight of the 23 insertions were disregarded as the likely result of assembly errors in *A. laevis*, as their flanking sequences were in the middle of two different contigs in both *E. ijimae* and *H. melanocephalus*. The flanking regions of the remaining 15 insertions were contiguous in *E. ijimae* and *H. melanocephalus*. We report these 15 insertions in table 2. We consider the insertion of RTE_Snek_2 into the 3ʹ UTR of the Adenylate Cyclase Type 4 (*ADCY4*) gene as the most interesting of these, as it is the only gene out the 15 that is present in a KEGG environmental adaptation pathway (circadian entrainment). However, testing the adaptive significance of these insertions will have to await improvement of the genome assembly and population genetic data for *A. laevis*. We note that many of these genes are likely to have pleiotropic effects as regulators of transcription or protein turnover, thus complicating future assessments of their adaptive significance. However, changes in pleiotropic genes have the potential to amplify adaptive changes in other loci (Østman et al. 2012).

## Discussion

We have identified seven LINE subfamilies present in sea snakes and one present in sea kraits, yet absent from their terrestrial relatives. The two competing hypotheses for this presence/absence pattern are loss from the terrestrial species or HTT to the marine species. If the seven subfamilies were lost from the terrestrial species, we would expect to see similar subfamilies still present in other squamates. Our search of 630 additional metazoans revealed the seven subfamilies to be absent not just from other squamates, but from all other tetrapods. For the majority of the seven subfamilies, the most similar LINE was present in a teleost fish, indicating either that the LINEs were repeatedly lost from all other tetrapods following their divergence from teleost fish 400 Ma, or the subfamilies were horizontally transferred into sea snakes and sea kraits following their divergence from terrestrial relatives.

Based on the observed patchy phylogenetic distribution, the high similarity of HTT TEs to those from distantly related marine species, and the discordance of the species and LINE phylogenies (figs. 3 and 5), the most parsimonious explanation is that the seven LINEs identified in *A. laevis* and one identified in *L. colubrina* were horizontally transferred from marine species following the transition of the ancestors of these snakes to a marine habitat. Additionally, the estimated timing of transfer supports independent transfers of both Rex1-Snek_1H2 and Rex1-Snek_1H3 into the *Aipysurus* and *Hydrophis* lineages (supplementary table 2, Supplementary material online). Although all seven LINE subfamilies are currently expressed in *A. laevis* based on transcriptome data, the number of large, near-identical fragments of RTE-Snek_1, RTE-Snek_2, and Proto2-Snek found within the *A. laevis* genome is larger than for the Rex1s. This indicates potentially greater replication of RTE-Snek_1, RTE-Snek_2, and Proto2-Snek since the HTT events in the past 3–17 Myr (Sanders et al 2008, 2012, 2013; Lee et al. 2016).

As all seven of the HTT LINE subfamilies are most similar to LINEs found in distantly related marine metazoans, we hypothesize that the donor species for each is likely a marine fish or invertebrate. However, the degree of sequence divergence between the LINE from *L. colubrina* and the seven LINEs from *A. laevis* from the most similar LINEs from aquatic species means we cannot identify a specific donor species. Likely donors and vectors of HTT are pathogens, predators, prey, parasites, and epibionts (Gilbert and Feschotte 2018). Sea snake diets vary greatly; some species are generalists that eat a wide variety of fish and occasionally crustaceans, cephalopods, and mollusks, whereas others specialize on burrowing eel-like or goby-like fish or feed exclusively on fish eggs (Sherratt et al. 2018). Parasites of sea snakes include isopods, nematodes, tapeworms, and flatworms, whereas epibionts include various, hydrozoans, polychaetes, decapods, gastropods, bivalves, and Bryozoa (Saravanakumar 2012; Gillett 2017). As very few species with ranges overlapping those of *Laticauda* and *Aipysurus* have been sequenced, and the range of *Aipysurus* spans highly biodiverse habitats, it is unlikely we will further narrow the donor of any of these eight LINE subfamilies without significant additional genome sequence data from Indo-West Pacific tropical marine species.

Although we were unable to identify specific donor species, our finding of HTT between marine species is in line with multiple past studies that reported HTT within and across marine phyla. HTT is prolific and particularly well described in aquatic microbial communities (reviewed in-depth in Sobecky and Hazen [2009]). HTT of LINEs, LTR retrotransposons and DNA transposons has been reported in marine metazoans, with past studies describing the transfer of Rex1s and Rex3s between teleost fishes (Volff et al. 2000; Carducci et al. 2018), *Steamer*-like LTR retrotransposons both within and across phyla (Metzger et al. 2018), L1 and BovB LINEs within and across phyla (Ivancevic et al. 2018), Mariner DNA transposons between diverse crustaceans (Casse et al. 2006), and a wide variety of TEs between tetrapods and teleost fish (Zhang et al. 2020). What sets our findings apart is that HTTs in this report have occurred multiple times as a result of the recent terrestrial to marine transition of the *Aipysurus/Hydrophis* common ancestor. The transfer of all seven LINEs occurred <18 Ma from aquatic animal donor species that diverged from snakes >400 Ma (Broughton et al. 2013; Hughes et al. 2018). As illustrated in figure 3, the varying presence/absence of the seven LINEs across the three species of sea snakes is indicative of nine independent HTT events as opposed to a single event. The recent timing of HTT into marine squamates is not specific to sea snakes, as we found transfer of an RTE-Kret to the sea kraits which underwent an independent transition to the marine habitat. These repeated invasions suggest aquatic environments potentially foster HTT, with more examples likely to be revealed by additional genome sequences from marine species.

The likely ongoing replication of all seven *A. laevis* HTT LINEs, as evidenced by both the presence of insertions and transcripts with near 100% identity, continues to contribute genetic material to the evolution of *Aipysurus*. Previous investigators have reported entire genes, exons, regulatory sequences, and noncoding RNAs in vertebrates derived from transposons, as well as TE insertions leading to genomic rearrangement (reviewed in-depth in Warren et al. [2015]). For snakes, Peng et al. (2020) described the expansion of LTR elements across *H. curtus* leading to adaptive changes in the marine environment. Similarly, the insertion of CR1 fragments near phospholipase A2 venom genes in vipers led to nonallelic homologous recombination, in turn causing duplication of these genes (Fujimi et al. 2002). Rapid genomic innovation would have been necessary for *Aipysurus* to adapt to the marine environment, with the independent evolution of paddle-like tails, salt excretion glands, and dermal photoreception following their divergence from their most recent common ancestor with *Hydrophis* (Brischoux et al. 2012; Sanders et al. 2012; Crowe-Riddell et al. 2019). Other adaptations are likely to have occurred or are occurring for sea snakes to conform to their marine habitat, as evolutionary transitions from terrestrial to marine habits entail massive phenotypic changes spanning metabolic, sensory, locomotor, and communication-related traits. Our finding that 15 genes, most with likely pleiotropic effects, contain HTT insertions and thus may have altered expression will require further investigation. One of these genes, ADCY4 is particularly interesting as it is part of the circadian entrainment pathway. Transition to a marine environment is likely to require altered sensitivity of the circadian entrainment pathway to environmental cues of light intensity and wavelength. Future research to examine the association between these HTT-derived sequences and adaptation will require investigation of differential regulation of these genes between terrestrial and marine snakes in a variety of tissues as well as improvement of the *A. laevis* genome assembly and collection of population genomic data.

## Conclusions

Our findings reveal repeated HTT of LINEs into fully marine and amphibious lineages of marine elapids as a result of their transition from a terrestrial environment. The HTT LINE insertions near genes and continued expression of all seven HTT LINE subfamilies is indicative of possible ongoing impact on the adaptive evolution of *Aipysurus*. Taken together, our results support a likely role for habitat transitions as direct contributors to the evolution of metazoan genomes, rather than solely acting through selection from altered environmental conditions.

## Materials and Methods

### Outline of Methods

Our study aimed primarily to identify TE subfamilies present in sea snakes yet absent from close terrestrial relatives, determine if their absence was due to TE loss or HTT, and if due to HTT find the potential donor or vector species. Our secondary aim was to determine if HTT subfamilies likely remain active in sea snakes based on transcriptomic data. Our final aim was to check if any HTT TE subfamilies discovered may have impacted the evolution of sea snakes since their divergence from terrestrial snakes by identifying insertions near/in genes and if these genes had roles in pathways important in adaptation to the marine habitat.

### Identification and Classification of Repetitive Sequences in *A. laevis*

We identified repetitive sequences present in the Ludington et al. (dx.doi.org/10.5281/zenodo.3975254) *A. laevis* assembly using CARP (Zeng et al. 2018). Using RPSTBLASTN 2.7.1+ (Marchler-Bauer and Bryant 2004) and a custom library of position-specific scoring matrices from the CDD and Pfam databases (Finn et al. 2016; Marchler-Bauer et al. 2017), we identified protein domains present in all consensus sequences over 800 bp in length found by CARP. Sequences were classified as potential LINEs, LTR retroelements and various DNA transposons based on the presence of relevant protein domains following the Wicker et al. (2007) classification. For example, we treated consensus sequences containing over 80% of both an exo-endonuclease domain and a reverse transcriptase domain as potential LINEs. For a full breakdown of protein domains used to classify retroelements, see supplementary table 3, Supplementary material online. We used CENSOR 4.2.29 (Kohany et al. 2006) to further classify the consensus sequences. To reduce redundancy, we aligned all potential TEs to all other potential TEs using BLASTN 2.7.1+ (Altschul et al. 1990; Camacho et al. 2009) with default parameters and removed consensus sequences with both 94% or higher pairwise identity to, and 50% or higher coverage by longer consensus sequences.

### Search for Ab Initio Annotated TEs in Close Terrestrial Relatives

To determine if the TEs subfamilies discovered were present in closely related species, we used megablast 2.7.1+ (Altschul et al. 1990; Camacho et al. 2009) to perform a nucleotide search for the consensus sequences of each subfamily in the genomes of two closely related terrestrial elapids (*N. scutatus* and *P. textilis*) (provided by Richard Edwards), and a more distantly related semi-marine elapid (*L. colubrina*) (Kishida et al. 2019). We treated all CARP sequences which were found by megablast in both *N. scutatus* and *P. textilis* as ancestrally shared, and all others as potential HTT candidates (all were LINEs). After discovering a highly similar subfamily was present in *L. colubrina* but absent from the two terrestrial snakes (RTE-Kret), we manually curated it using a “search, extend, align, trim” method adapted from Platt et al. (2016) and Suh et al. (2018) (see supplementary Methods, Supplementary material online and description below).

### Curation of TEs Absent from Close Terrestrial Relatives

To create a better consensus for each LINE subfamily, we manually curated new consensus sequences using a “search, extend, align, trim” method (explained in greater detail in supplementary Methods, Supplementary material online, script at https://github.com/jamesdgalbraith/HT_Workflow/blob/master/PresenceAbsence/extendAlignSoloRstudio.R, last accessed September 16, 2020). We used megablast 2.7.1+ (Altschul et al. 1990; Camacho et al. 2009) to search for the consensus sequence of a subfamily within the *A. laevis* genome. We selected the 25 best hits over 1,000 bp based on bitscore and extended the coordinates of these sequences by 1,000 bp at each end of the hit. We constructed multiple sequence alignments (MSAs) of the extended sequences using MAFFT v7.310 (Katoh and Standley 2013). Where multiple full-length sequences showing significant lack of homology were present, the LINE subfamily was split into multiple subfamilies (see supplementary fig. 9, Supplementary material online). Finally, we manually edited the extended sequences in Geneious Prime 2020.0.2 to remove nonhomologous regions and created a new consensus sequence. If only one full-length copy of a subfamily was present in the genome, it was used instead of a consensus sequence. We used PCOILs (Gruber et al. 2006) and HHpred (Zimmermann et al. 2018) searches of the translated Open Reading Frames (ORFs) against the CDD and Pfam databases (Finn et al. 2016; Marchler-Bauer et al. 2017) to identify any additional protein domains or structures present in the seven LINEs.

### Search for HTT Candidate LINEs in the Genomes and Transcriptomes of Other Sea Snakes

Similar to the search of closely related terrestrial species, we used megablast to perform reciprocal searches for the consensus sequences of the seven *Aipysurus* LINE subfamilies in the genomes of *H. melanocephalus* and *Emydocephalus annulatus* (Kishida 2019), and assembled transcriptomes from various tissues of *A. laevis*, *A. tenuis*, and *H. major* from Crowe-Riddell et al. (2019).

### Estimating Timing of HTT Events by Substitution Rate

We estimated the timing of the seven HTT events using a custom R script (https://github.com/jamesdgalbraith/HT_Workflow/blob/master/Divergence/insertion_time_calculator.R, last accessed September 16, 2020). We identified all copies of the seven *A. laevis* HTT subfamilies in the *A. laevis* assembly using megablast. A reciprocal megablast search using the identified copies was carried out against the seven *A. laevis* HTT subfamily consensus sequences to identify the most similar sequence based on pairwise identity. Using the reciprocal megablast search output, we calculated the mean substitutions per site for each HTT subfamily. Finally, using an elapid whole-genome substitution rate estimate from Ludington and Sanders (under review by *Molecular Ecology*) of 1.25 × 10e−08 per site per generation and a generation time of 10 years, we calculated the HTT event timing of each subfamily (supplementary table 2, Supplementary material online).

### Search for and Curation of Similar TEs in Other Metazoan Genomes

To identify other species containing the seven *Aipysurus* and one *Laticauda* LINE subfamilies, we used the HTT LINE consensus sequences for BLASTN searches in of over 630 metazoan genomes downloaded from GenBank (Benson et al. 2017) using relaxed parameters (-evalue 0.00002 -reward 3 -penalty -4 -xdrop_ungap 80 -xdrop_gap 130 -xdrop_gap_final 150 -word_size 10 -dust yes -gapopen 30 -gapextend 6). We treated species containing a hit of at least 1,000 bp as potentially containing a similar LINE subfamily. From the BLASTN hits from these species, we attempted to manually curate subfamilies using a variant of the “search, extend, align, trim” method described in the supplementary Methods, Supplementary material online. If only one copy of the LINE subfamily was present in a genome assembly we did not include that species in the list of species containing similar LINEs in order to reduce false positives. We used a consensus sequence derived from the initial hits within the species as the query for the BLASTN search of the genome, and extended hits by 3,000 bp in the 5ʹ and 3ʹ directions. As illustrated in supplementary figure 9, Supplementary material online, if an MSA appeared to contain multiple LINE subfamilies, as judged by lack of sequence homology or gaps, it was split and consensuses were constructed for each individual family. As homologous, yet highly diverged, Rex1 and RTE subfamilies were identified in other elapids we used the same “search, extend, align, trim” method to curate the most similar repeats in the *A. laevis* assembly, using the consensus from *N. scutatus* as the initial query. All subfamilies identified in *N. scutatus* had highly similar homolog in *A. laevis*.

### Characterizing Divergence Patterns in the HT Repeats across Hydrophiinae

To identify fragments of the seven *Aipysurus* and one *Laticauda* HTT LINE subfamilies and determine their divergence from the consensus sequences, we performed a reciprocal best hit search using BLASTN 2.7.1+ (Altschul et al. 1990; Camacho et al. 2009) on the *A. laevis*, *E. ijimae*, *Hydrophis cyanocinctus*, *H. melanocephalus*, *N. scutatus*, *P. textilis*, *L. colubrina*, and *O. hannah* assemblies. HTT consensus sequences were used as the initial query, with resulting hits then used as queries against a database containing the original consensus sequences.

### Repeat Phylogeny Construction

For constructing repeat phylogenies, we created two libraries; one containing all Rex1s we curated and Rex1s derived from Repbase; and another containing all RTEs, we curated and all RTE-like (Proto2, RTE, and BovB) sequences from Repbase. In addition, each library contained an outgroup LINE based on the Eickbush and Malik (Eickbush and Malik 2002) phylogeny of LINEs. We removed all sequences not containing at least 80% of both the endonuclease and reverse transcriptase domains from each library based on RPSTBLASTN (Marchler-Bauer and Bryant 2004) searches against the NCBI CDD (Marchler-Bauer et al. 2017).

We created nucleotide MSAs of each library of LINEs using MAFFT v7.310 (Katoh and Standley 2013) and removed poorly aligned regions using Gblocks (Talavera and Castresana 2007) allowing smaller final blocks, gap positions within the final blocks and less strict flanking positions. Finally, we constructed phylogenies from the trimmed MSA using RAxML (Stamatakis 2014) with 20 maximum likelihood trees and 500 bootstraps.

### Species Phylogeny Construction

We used TimeTree (Hedges et al. 2006) to infer species phylogenies presented in figure 4. In cases in which a species of interest was not present in the TimeTree database, where possible we used an appropriate species from the same clade in its place and corrected the species names on the resulting tree.

### Repeat Insertions Near and in Genes

Using the plyranges (Lee et al. 2019) and GenomicRanges R packages (Lawrence et al. 2013) (53, 54), we identified any insertions of the HTT LINEs into coding exons, UTRs and upstream of 5ʹ UTRs for gene annotations from Ludington et al. (https://dx.doi.org/10.5281/zenodo.3975254, last accessed September 16, 2020) (https://github.com/jamesdgalbraith/HT_Workflow/blob/master/GeneInteraction/overlapSearch.R, last accessed September 16, 2020).

To confirm that insertions were assembled correctly, we used BLASTN to search for the repeats extended by 2,000 bp in each direction in the *E. ijimae* and *H. melanocephalus* assemblies. We selected the best hits from each species based on query coverage and percent identity. Using MAFFT v7.310 (Katoh and Standley 2013), we constructed MSAs of each extended repeat and the corresponding regions from the two other assemblies (https://github.com/jamesdgalbraith/HT_Workflow/blob/master/GeneInteraction/insertionConfirmation.R, last accessed September 16, 2020). By manually viewing the resulting alignment in Geneious and the raw BLASTN output, we determined if the repeat insertions were assembled correctly. To confirm the insertion of RTE-Snek_2 identified in the 3ʹ UTR of *ADCY4*, we perform megablast searches of the *A. laevis* transcriptome from Ludington et al. (https://dx.doi.org/10.5281/zenodo.3993854, last accessed September 16, 2020).

## Supplementary Material

Supplementary data are available at *Genome Biology and Evolution* online.

## Supplementary Material

evaa208_Supplementary_Data
