# Supplementary material for: New Environment, New Invaders—Repeated Horizontal Transfer of LINEs to Sea Snakes
Source: Genome Biol Evol. 2020 Oct 6;12(12):2370–83. doi: 10.1093/gbe/evaa208 (PMC7846101; doi:10.1093/gbe/evaa208)
Supplement: evaa208_Supplementary_Data [file evaa208_supplementary_data.zip › GBE_Sea_Snake_Draft_SI_R2_clean.docx]

Supplementary Information for

New environment, new invaders - repeated horizontal transfer of LINEs to sea snakes

James D. Galbraith^1^, Alastair J. Ludington^1^, Alexander Suh^2,3^, Kate L. Sanders^1^, David L. Adelson*^1^

1) School of Biological Sciences, University of Adelaide, Adelaide, SA 5005, Australia

2) Department of Ecology and Genetics - Evolutionary Biology, Evolutionary Biology Centre, Uppsala University, SE-752 36 Uppsala, Sweden

3) Department of Organismal Biology - Systematic Biology, Evolutionary Biology Centre, Uppsala University, SE-752 36 Uppsala, Sweden

* David L. Adelson^1^

Email: david.adelson@adelaide.edu.au

**This PDF file includes:**

SI Figures 1-11

SI Tables 1-3

Legends for SI Datasets 1-9

**Other supplementary materials for this manuscript include the following:**

Datasets S1 to S8

**Supplementary Methods and Results**

**Detailed description of “search, extend, align, trim” curation method**

Through the manuscript we have curated consensus sequences of LINE subfamilies using a method described as a “search, extend, align, trim” method. We first searched a genome assembly for similar repeats using megablast with default parameters (when searching highly similar repeats in the other elapid species), or blastn with the custom parameters -evalue 0.00002 -reward 3 -penalty -4 -xdrop_ungap 80 -xdrop_gap 130 -xdrop_gap_final 150 -word_size 10 -dust yes -gapopen 30 -gapextend 6 when performing the search of all 630 species.. After filtering out hits less than 1000bp we selected the top 20 hits based on bitscore, and extended the coordinates 500bp in either direction at each end, aligned the sequences using MAFFT with the “--localpair” parameter ([Katoh and Standley, 2013](https://www.zotero.org/google-docs/?vHUX4i)). When viewing the alignments in Geneious if subfamilies were apparent multiple we split the MSA into separate alignments (see SI Fig. 1). If the sequences aligned well across the MSA we created a consensus sequence and repeated the process using megablast. We adjusted the distance the sequences were extended until the MSA showed significant discordance at each end, suggesting the entire transposons were found. For LTR retrotransposons and DNA transposons we deleted the discordance at each and constructed a consensus. As LINEs show a 5’ truncation pattern we removed the discordance from the 5’ end of each individual sequence before constructing a consensus. We constructed all consensuses in Geneious using a threshold of 50%. All of our BLAST searches used BLAST+ 2.7.1 ([Altschul](https://www.zotero.org/google-docs/?e37IQt) et al., 1990).

When manually curating sequences found in species outside of elapids we determined if hits were not assembly errors or contamination by examining the number of hits BLAST found of each sequence. If less than three copies of the TE of any length could be found we considered the hit to be either an assembly error or contamination.


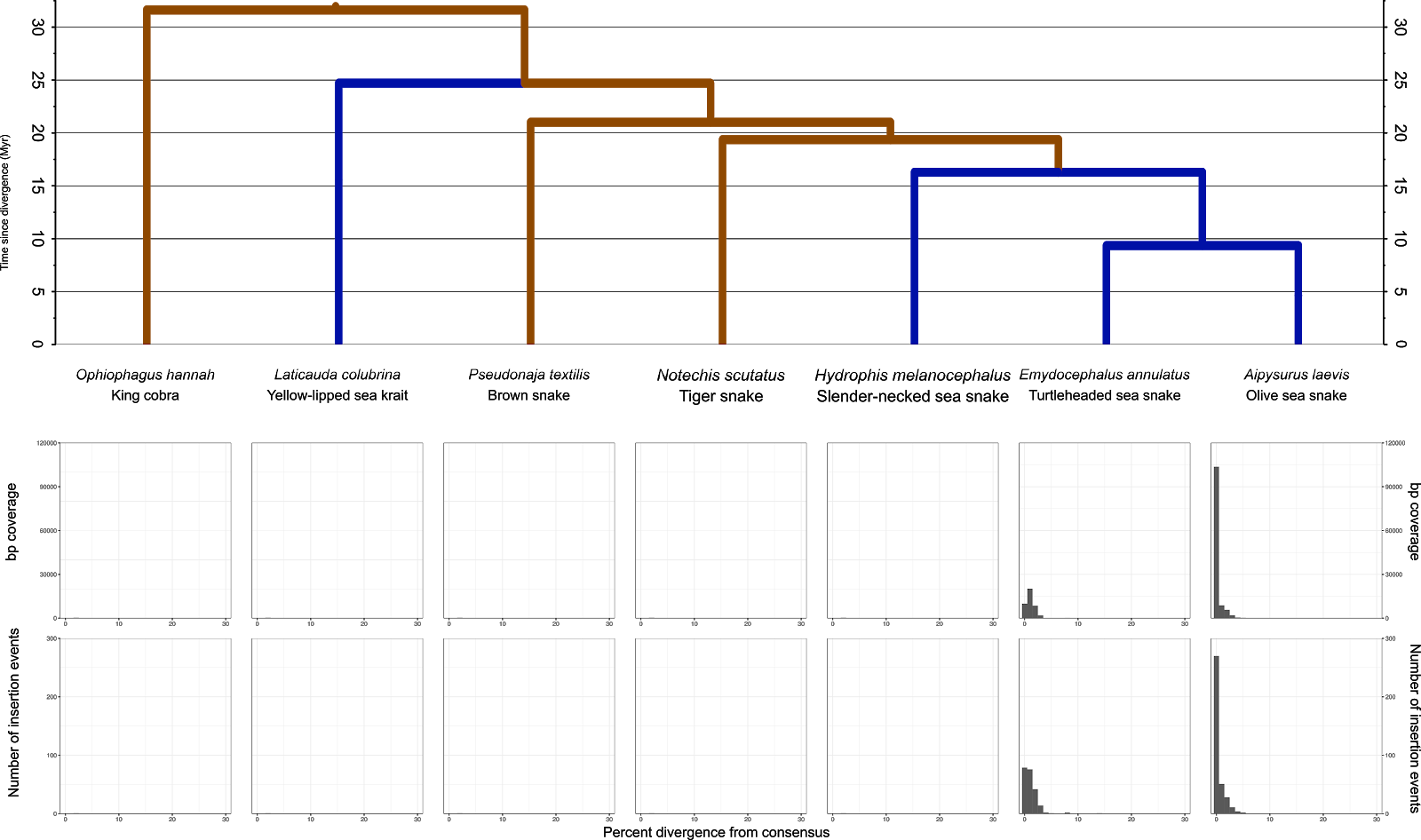


**SI Figure 1.** Number of insertions using fragments as a proxy, and bp coverage vs divergence from consensus of Proto2-Snek insertions identified in *Ophiophagus hannah*, *Laticauda colubrina* and six Hydrophiinae. Presence of LINEs detected using BLASTN+ 2.7.1 [(Altschul et al. 1990; Camacho et al. 2009)](https://www.zotero.org/google-docs/?ZzFKhD) and plotted in RStudio [(RStudio Team 2015)](https://www.zotero.org/google-docs/?2JxMZf) using ggplot2 [(Wickham 2011)](https://www.zotero.org/google-docs/?miGuAP).


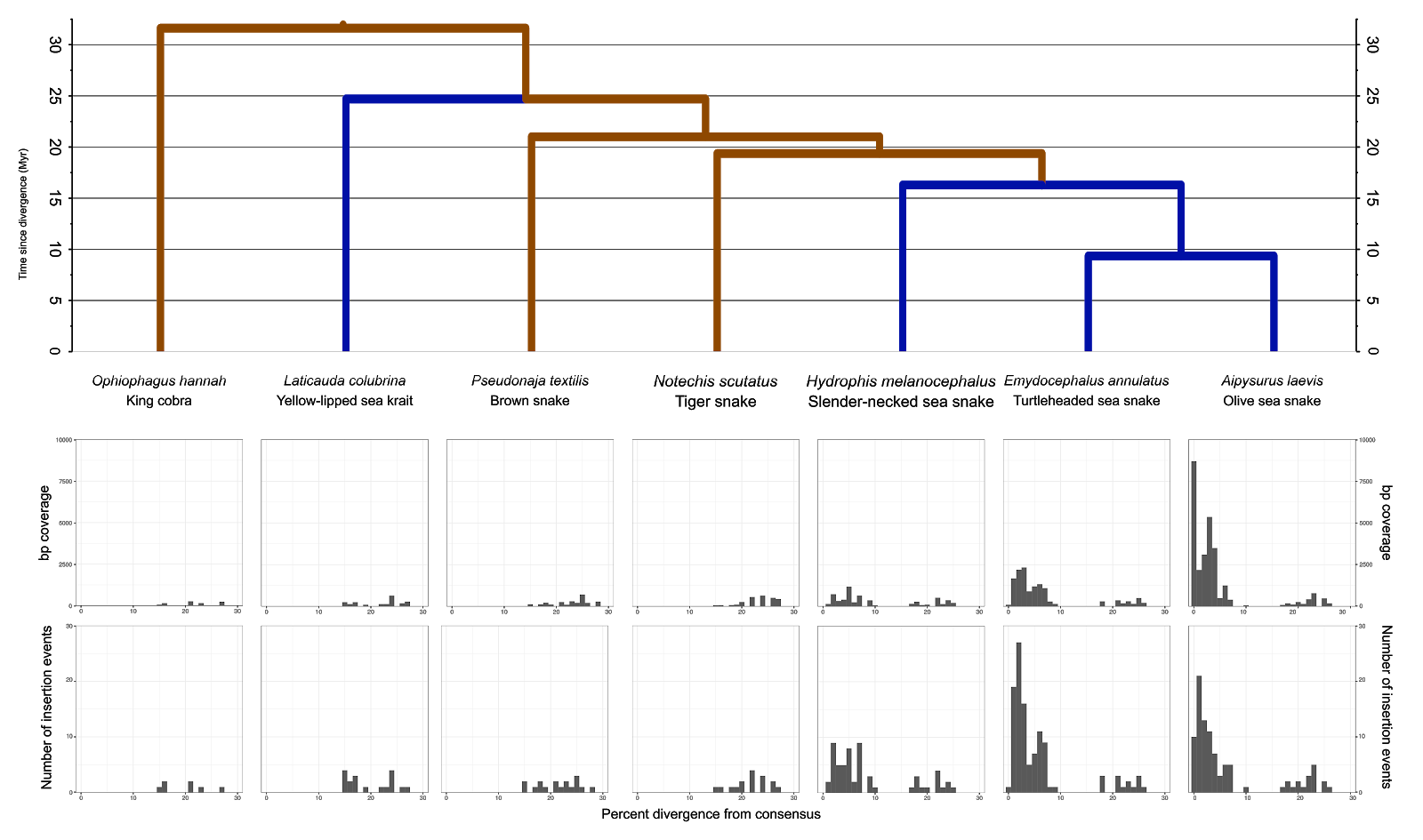


**SI Figure 2.** Number of insertions using fragments as a proxy, and bp coverage vs divergence from consensus of Rex1-Snek_1H1 insertions identified in *Ophiophagus hannah*, *Laticauda colubrina* and six Hydrophiinae. Note that insertions with >15% divergence represent similarity to distant, ancestral Rex1 elements found in non-avian reptiles. Presence of LINEs detected using BLASTN+ 2.7.1 [(Altschul et al. 1990; Camacho et al. 2009)](https://www.zotero.org/google-docs/?7z2qee) and plotted in RStudio [(RStudio Team 2015)](https://www.zotero.org/google-docs/?NmeEiW) using ggplot2 [(Wickham 2011)](https://www.zotero.org/google-docs/?Y28w90).


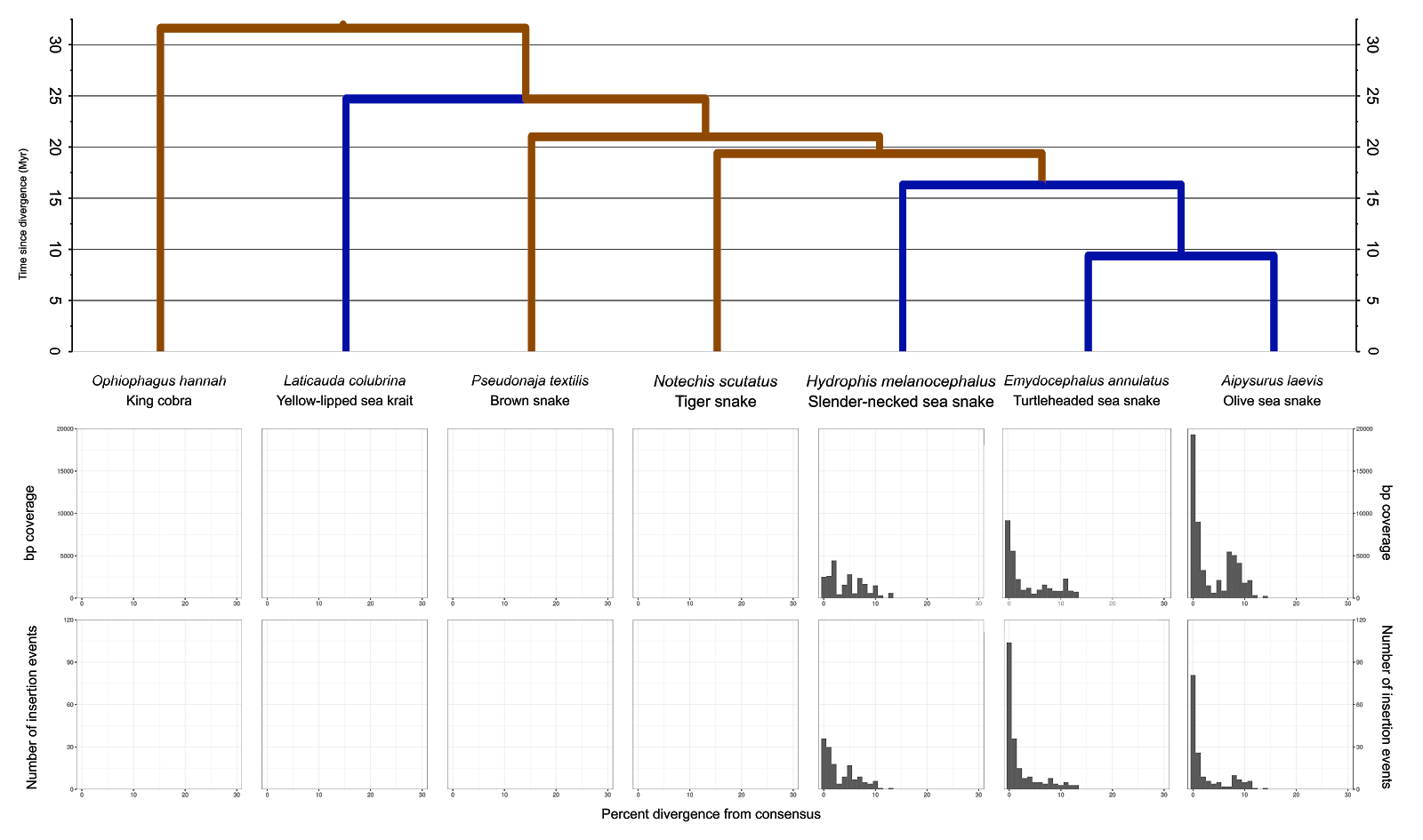


**SI Figure 3.** Number of insertions using fragments as a proxy, and bp coverage vs divergence from consensus of Rex1-Snek_2 insertions identified in *Ophiophagus hannah*, *Laticauda colubrina* and six Hydrophiinae. Presence of LINEs detected using BLASTN+ 2.7.1 [(Altschul et al. 1990; Camacho et al. 2009)](https://www.zotero.org/google-docs/?5q2Tvk) and plotted in RStudio [(RStudio Team 2015)](https://www.zotero.org/google-docs/?aoAdAx) using ggplot2 [(Wickham 2011)](https://www.zotero.org/google-docs/?mQ0RyE).


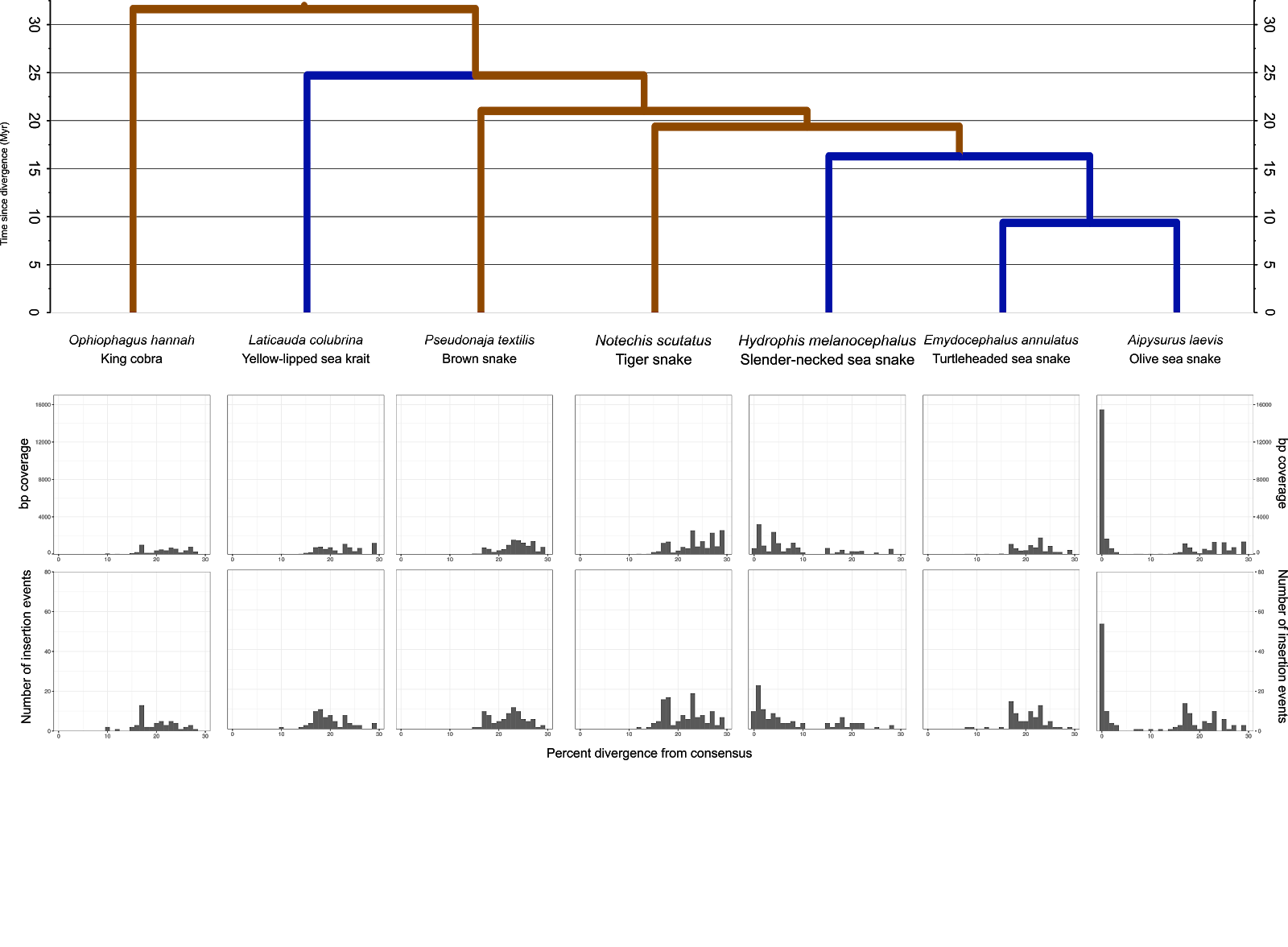


**SI Figure 4.** Number of insertions using fragments as a proxy, and bp coverage vs divergence from consensus of Rex1-Snek_1H2 insertions identified in *Ophiophagus hannah*, *Laticauda colubrina* and six Hydrophiinae. Note that insertions with >15% divergence represent similarity to distant, ancestral Rex1 elements found in non-avian reptiles. Presence of LINEs detected using BLASTN+ 2.7.1 [(Altschul et al. 1990; Camacho et al. 2009)](https://www.zotero.org/google-docs/?VEQUIN) and plotted in RStudio [(RStudio Team 2015)](https://www.zotero.org/google-docs/?GGjfXx) using ggplot2 [(Wickham 2011)](https://www.zotero.org/google-docs/?DJw3kw).


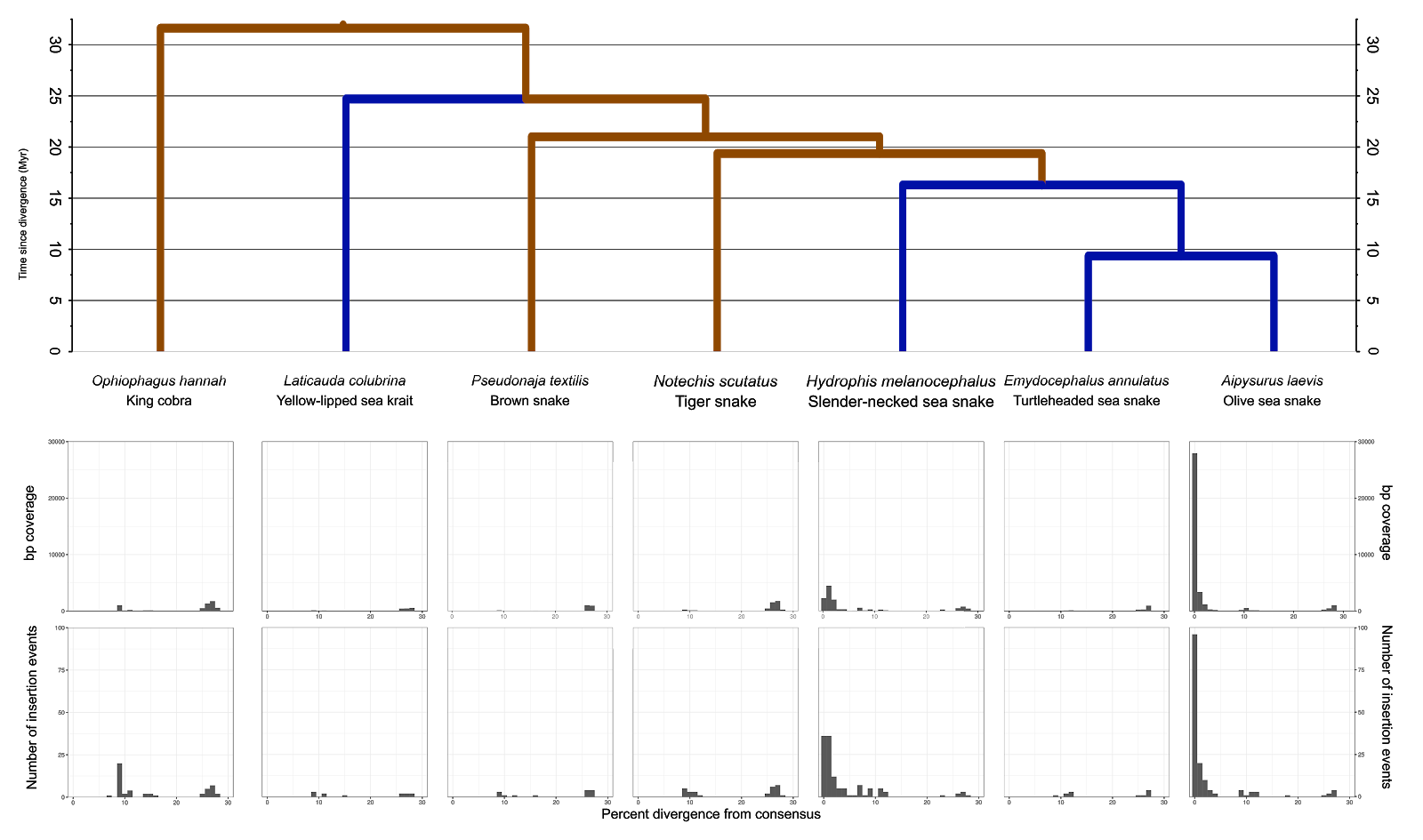


**SI Figure 5.** Number of insertions using fragments as a proxy, and bp coverage vs divergence from consensus of Rex1-Snek_1H3 insertions identified in *Ophiophagus hannah*, *Laticauda colubrina* and six Hydrophiinae. Note that insertions with >15% divergence represent similarity to distant, ancestral Rex1 elements found in non-avian reptiles. Presence of LINEs detected using BLASTN+ 2.7.1 [(Altschul et al. 1990; Camacho et al. 2009)](https://www.zotero.org/google-docs/?N3TXC1) and plotted in RStudio [(RStudio Team 2015)](https://www.zotero.org/google-docs/?XXn0sp) using ggplot2 [(Wickham 2011)](https://www.zotero.org/google-docs/?r32MeY).


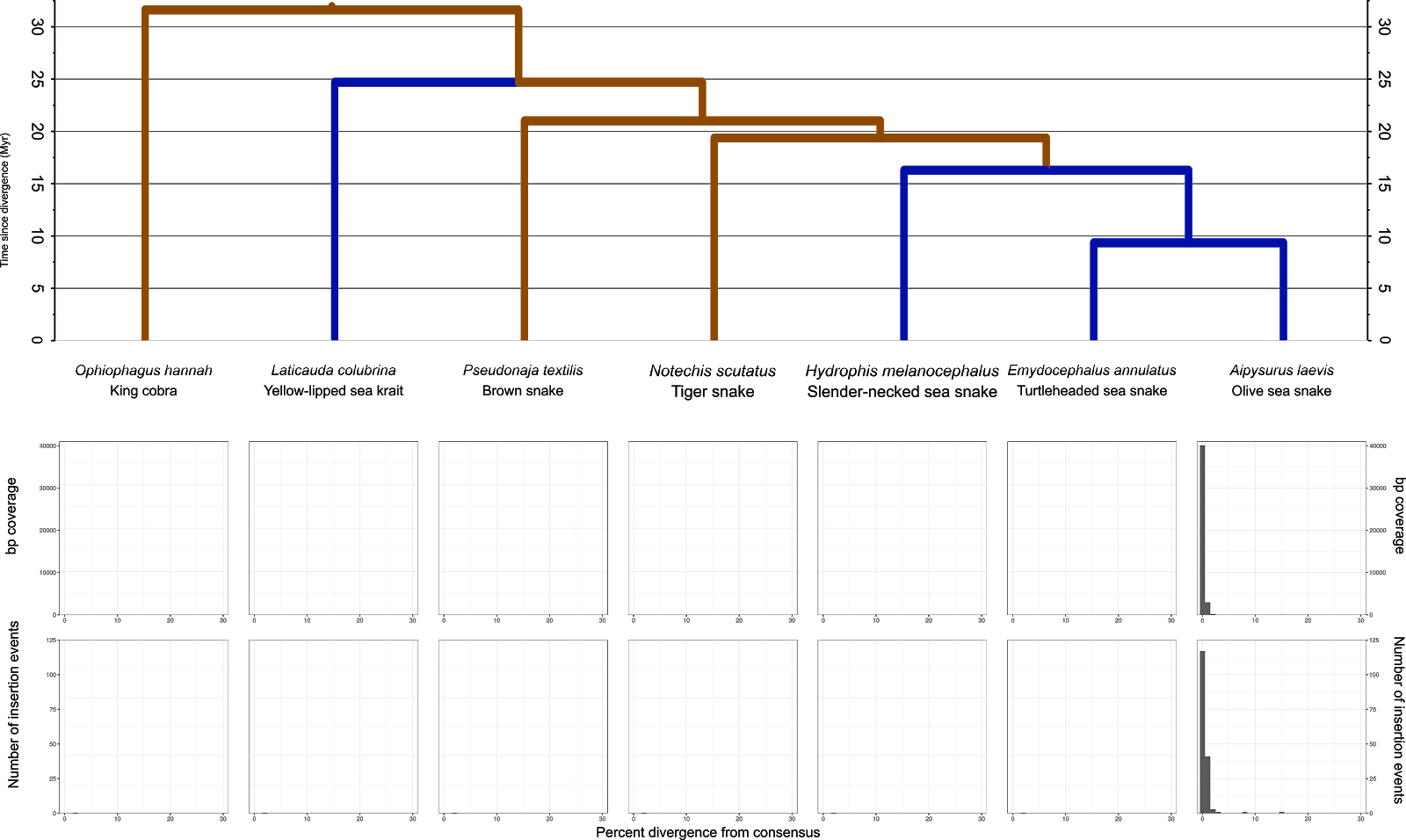


**SI Figure 6.** Number of insertions using fragments as a proxy, and bp coverage vs divergence from consensus of RTE-Snek_1 insertions identified in *Ophiophagus hannah*, *Laticauda colubrina* and six Hydrophiinae. Presence of LINEs detected using BLASTN+ 2.7.1 [(Altschul et al. 1990; Camacho et al. 2009)](https://www.zotero.org/google-docs/?9G6MKr) and plotted in RStudio [(RStudio Team 2015)](https://www.zotero.org/google-docs/?ARtPA1) using ggplot2 [(Wickham 2011)](https://www.zotero.org/google-docs/?MYjwR7).


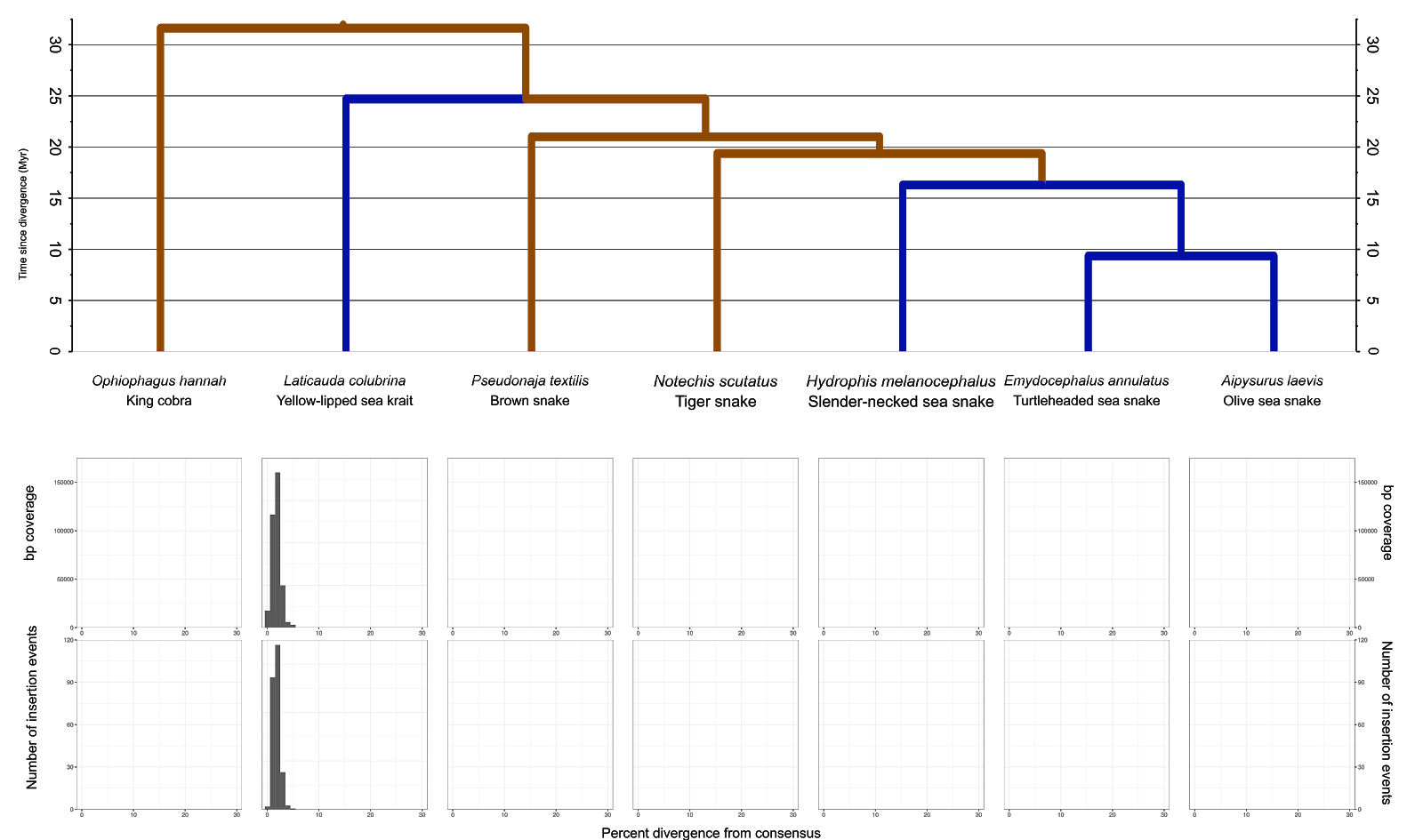


**SI Figure 7.** Number of insertions using fragments as a proxy, and bp coverage vs divergence from consensus of RTE-Kret insertions identified in *Ophiophagus hannah*, *Laticauda colubrina* and six Hydrophiinae. Presence of LINEs detected using BLASTN+ 2.7.1 [(Altschul et al. 1990; Camacho et al. 2009)](https://www.zotero.org/google-docs/?48FAzI) and plotted in RStudio [(RStudio Team 2015)](https://www.zotero.org/google-docs/?sf6F34) using ggplot2 [(Wickham 2011)](https://www.zotero.org/google-docs/?AsOWxG).


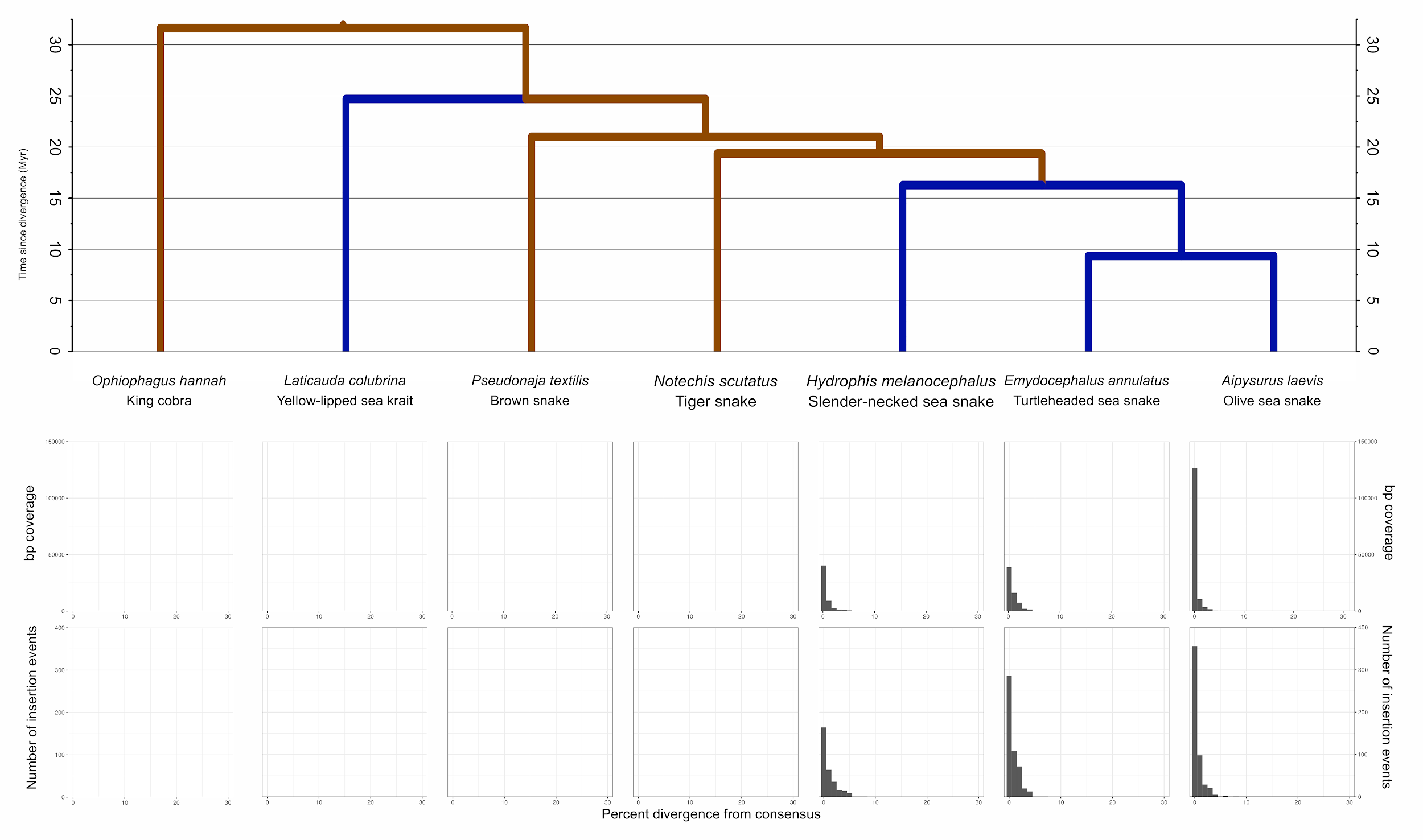


**SI Figure 8.** Number of insertions using fragments as a proxy, and bp coverage vs divergence from consensus of RTE-Snek_2 insertions identified in *Ophiophagus hannah*, *Laticauda colubrina* and six Hydrophiinae. Presence of LINEs detected using BLASTN+ 2.7.1 [(Altschul et al. 1990; Camacho et al. 2009)](https://www.zotero.org/google-docs/?wbdjQY) and plotted in RStudio [(RStudio Team 2015)](https://www.zotero.org/google-docs/?J0YkAd) using ggplot2 [(Wickham 2011)](https://www.zotero.org/google-docs/?9ohK9B).


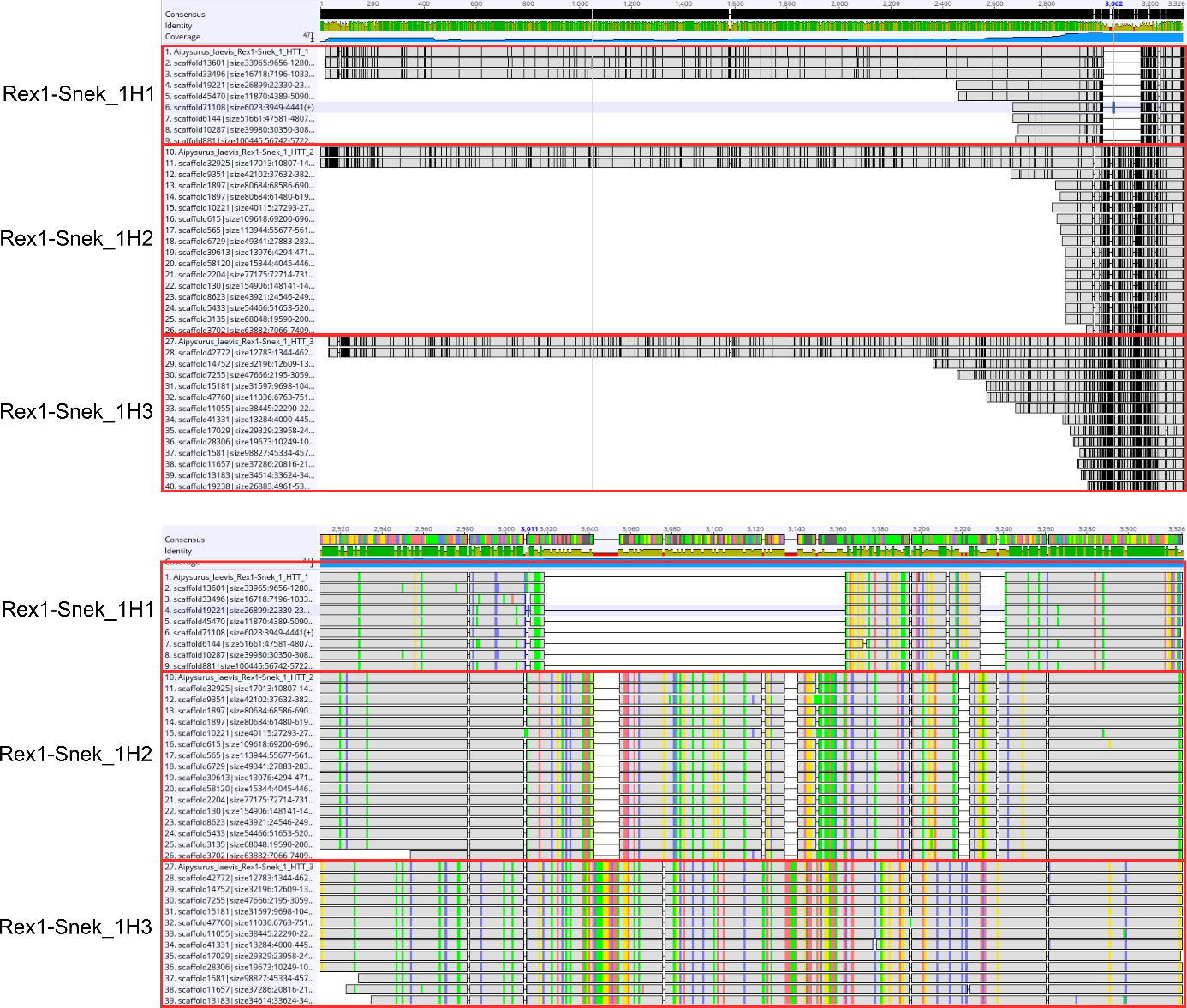


**SI Figure 9.** Multiple sequence alignments (MSA) of 40 Rex1-Snek_1H1, Rex1-Snek_1H2 and Rex1-Snek_1H3 sequences present in the *Aipysurus laevis* assembly and the consensus sequences constructed for each subfamily. The sequences were initially identified using a megablast search of the Rex1-Snek_1 sequence against the initial *Aipysurus laevis* genome assembly. This serves an illustration as how MSAs of LINEs identified in other species were split into separate subfamilies when necessary during the “search, extend, align, trim” method.


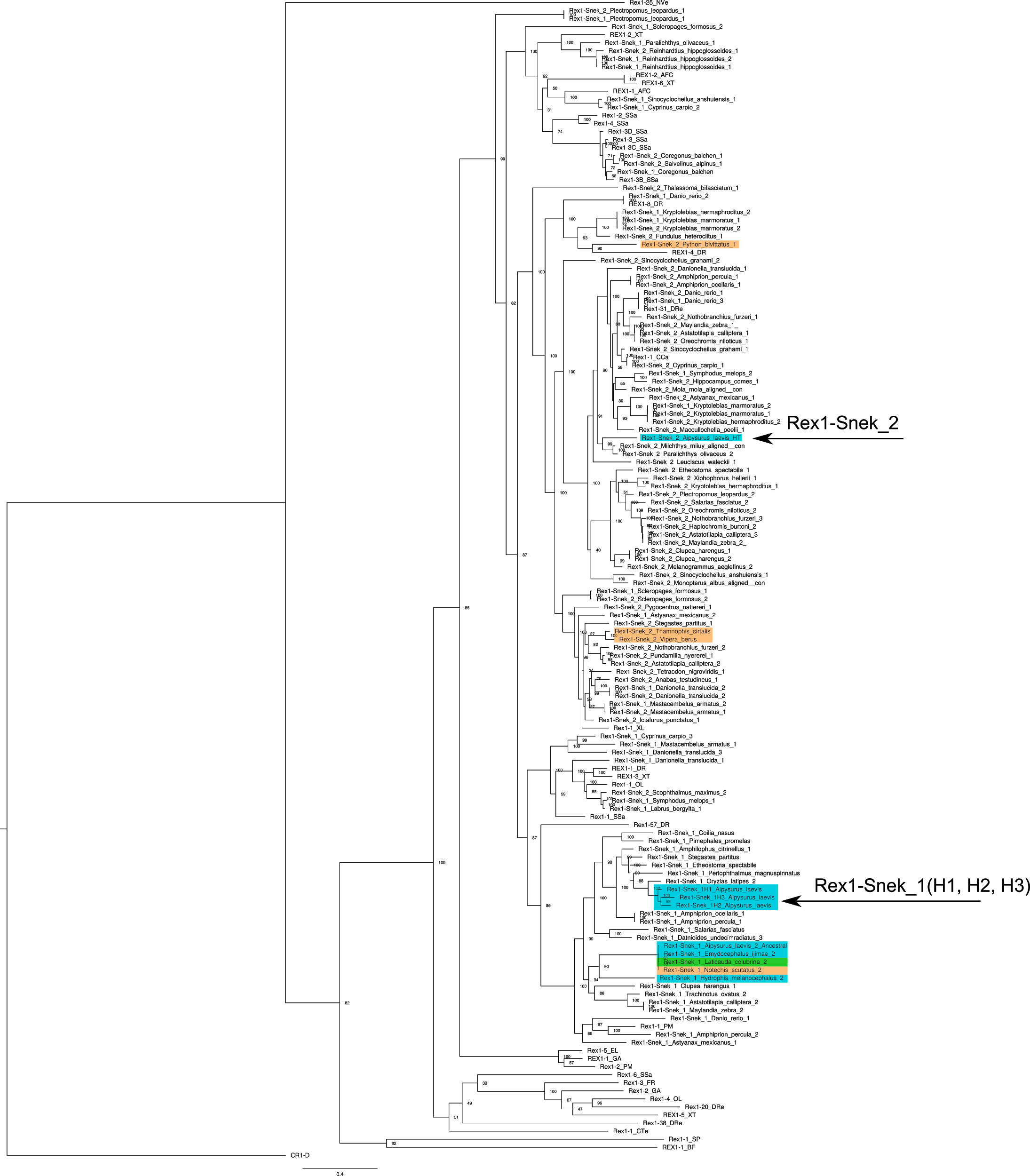


**SI Figure 10.** Full tree of all curated and Repbase Rex1-like LINEs containing both an endonuclease and a reverse transcriptase domain. Sequences found in sea snakes are highlighted in blue, sequences found in terrestrial snakes are highlighted in brown and sequences found in sea kraits are highlighted in green. Phylogeny created using RaxML (Stamatakis, 2014) from a multiple nucleotide sequence alignment generated using MAFFT (Katoh and Standley, 2013) and trimmed using Gblocks (Talavera and Castresana, 2007). Sequences available in SI Dataset 6, Newick tree in SI Dataset 7.


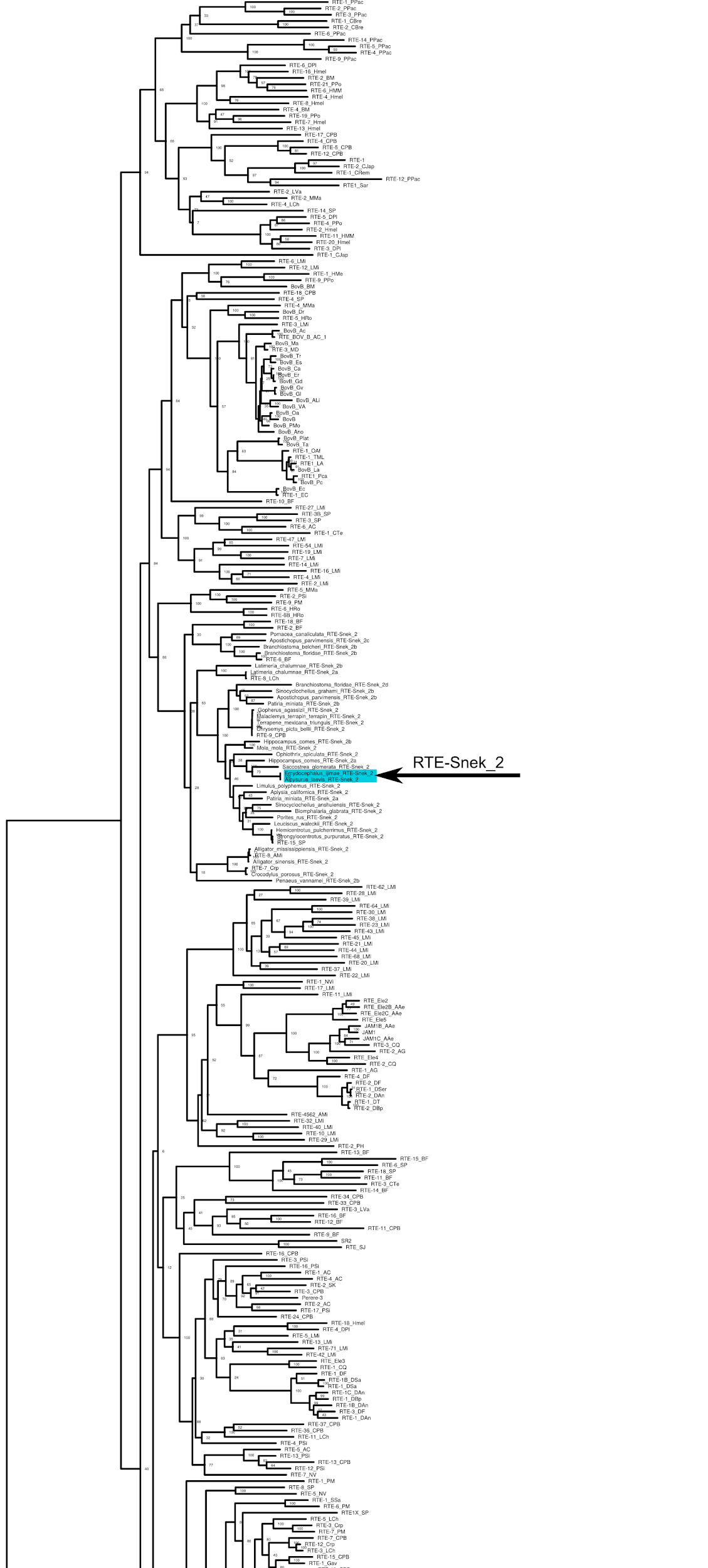

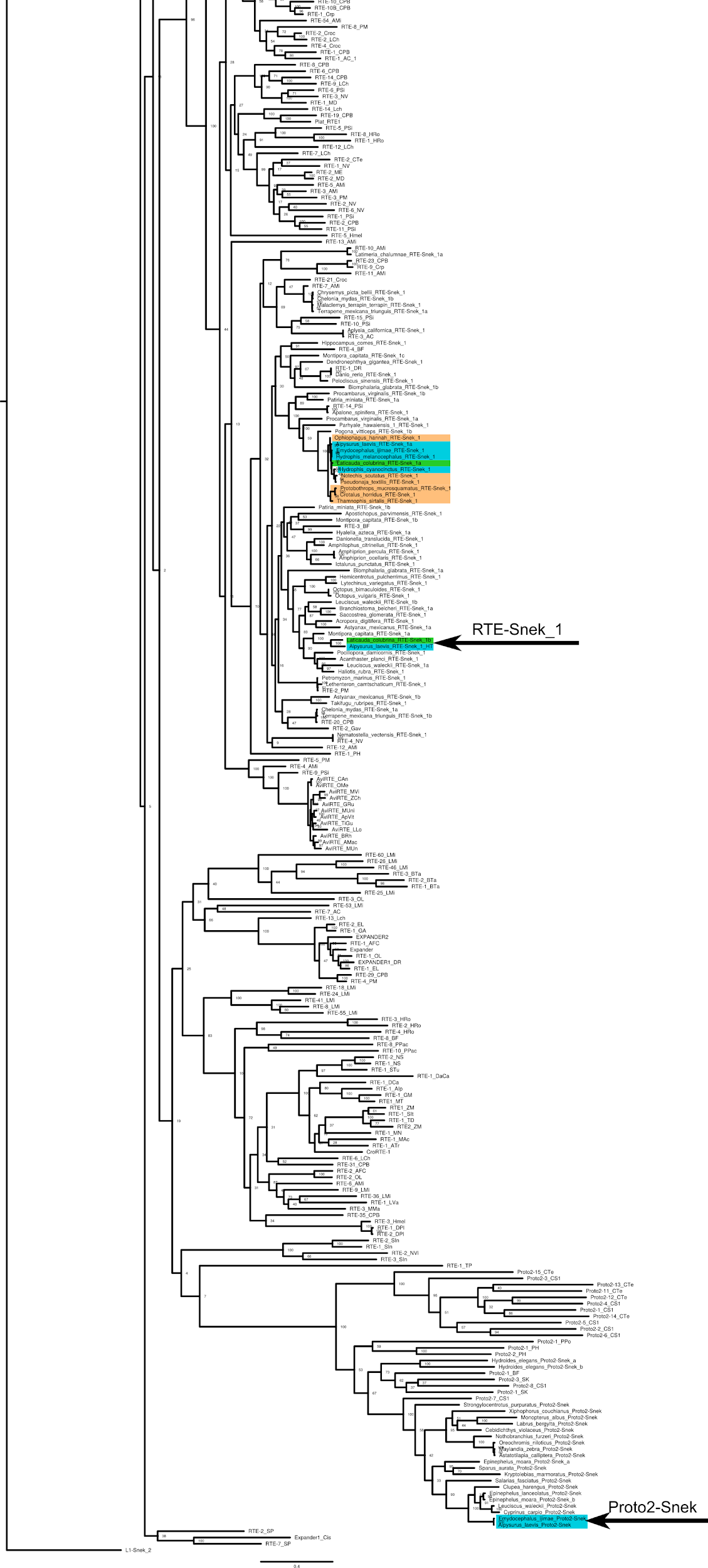


**SI Figure 11.** Full tree of all curated and Repbase RTEs and Proto2s containing both an endonuclease and a reverse transcriptase. Sequences found in sea snakes are highlighted in blue, sequences found in terrestrial snakes are highlighted in brown and sequences found in sea kraits are highlighted in green. Phylogeny created using RaxML (Stamatakis, 2014) from a multiple nucleotide sequence alignment generated using MAFFT (Katoh and Standley, 2013) and trimmed using Gblocks (Talavera and Castresana, 2007). Sequences available in SI Dataset 6, Newick tree in SI Dataset 8.

|  | HTT LINEs | All LINEs |
| --- | --- | --- |
| Total insertions | 1478 | 139942 |
| Insertions on same scaffold as genes | 536 | 43033 |
| Total Insertions into genes | 76 | 8491 |
| Insertions into coding exons | 1 | 725 |
| Insertions into UTRs | 2 | 572 |
| Insertions within 5000bp of 5’ UTR | 22 | 4022 |

**SI Table 1.** Raw number of all LINE insertions and HTT LINE insertions into *A. laevis* genome, exons and upstream of 5’UTRs with a divergence from consensus below 10%. Insertions detected using an intersect of the genome annotation and RepeatMasker repeat annotation of the *A. laevis* assembly from Ludington et al. (dx.doi.org/10.5281/zenodo.3975254) in RStudio [(RStudio Team 2015)](https://www.zotero.org/google-docs/?IFl3TN) using GenomicRanges and plyranges [(Lawrence et al. 2013; Lee et al. 2019)](https://www.zotero.org/google-docs/?KlKM9J).

| LINE Subfamily | Estimated HTT timing (MYA) | **Precise species divergence time for this event (MYA)**  **(Sanders et al. 2008,09,13)** | **Looser species divergence time for this event (MYA)**  **(Lee et al. 2016)** | Standard deviation (MYA) | Number of insertion events over 100bp |
| --- | --- | --- | --- | --- | --- |
| Proto2-Snek | 4.47 | 6.5-8.5 | 10-16 | 6.25 | 285 |
| Rex1-Snek_1H1 | 14.24 | 8.5-9.5 | 16-20 | 11.01 | 33 |
| Rex1-Snek_1H2 | 1.58 | 0-1.8 | 0-10 | 2.45 | 49 |
| Rex1-Snek_1H3 | 3.02 | 0-1.8 | 0-10 | 3.65 | 90 |
| Rex1-Snek_2 | 11.88 | 8.5-9.5 | 16-20 | 20.20 | 97 |
| RTE-Snek_1 | 2.65 | 0-1.8 | 0-10 | 2.81 | 71 |
| RTE-Snek_2 | 2.12 | 8.5-9.5 | 16-20 | 2.95 | 297 |

**SI Table 2.** Estimated timing of HTT events of 7 LINE subfamilies into *Aipysurus laevis* based on whole genome substitution rate from Ludington and Sanders (under review). We identified TEs and calculated the number of mismatches using megablast (Altschul et al., 1990), and estimated the divergence time in RStudio ([RStudio Team. 2015](https://www.zotero.org/google-docs/?e37IQt)).

| **TE Subclass/Family** | **Proteins domain PSSMs from CDD** |
| --- | --- |
| LINEs | **Reverse transcriptases:** RT_like, RT_nLTR_like, RVT_1, RT_G2_intron, RVT_1, TERT |
|  | **Exo-endonucleases:** EEP, EEP-2, Exo_endo_phos, Exo_endo_phos_2, L1-EN, R1-I-EN |
| Penelopes | **Reverse transcriptases:** RT_like, RT_nLTR_like, RVT_1, RT_G2_intron, RVT_1, TERT |
|  | **GIY-YIG endonuclease:** GIY-YIG_PLEs |
| ERVs | **Envelopes:** Ebola_HIV-1-like_HR1-HR2, Ebola_RSV-like_HR1-HR2, Ebola-like_HR1-HR2, HIV-1-like_HR1-HR2, GP41, ENVV1-like_HR1-HR2, HERV-Rb-like_HR1-HR2, HTLV-1-like_HR1-HR2, RSV-like_HR1-HR2, TLV_coat |
| Copias | **Integrase:** rve |
|  | **Reverse transcriptase**: RVT_2 |
|  | **RNase:** RNase_HI_RT_Ty1 |
| DIRs/Gypsys/Bel | **RNase:** RT_RNaseH_2, RT_RNaseH, RNase_HI_RT_Ty3, RNase_HI_RT_DIRS1, RNase_H_like |
|  | **Reverse transcriptase:** RT_ZFREV_like, RT_Rtv, RT_LTR, RT_DIRS1, RVT_1, RT_like |
|  | **Integrases:** Integrase_H2C2, zf-H2C2 |
| hAT DNA Transposons | **Transposase:** Dimer_Tnp_hAT |
| Tc/Mariner Transposons | **Helix-turn-helix:** HTH_Tnp_Tc3_2, HTH_Tnp_Tc5, HTH_Tnp_1, CENPB, COG3415, HTH_32, HTH_ARSR, HTH_28, HTH_38, HTH_7, HTH_48, HTH_23, HTH_24 |
|  | **DDEs:** DDE_1, DDE_3, Transposase_1, BrkDBD, COG3335 |
| PIF/Harbingers | **DNA binding domains:** Myb_DNA-bind_4, GT1 |
|  | **Transposases:** Plant_tran, DDE_Tnp_1, DDE_Tnp_4 |
| piggyBacs | **Transposase:** DDE_Tnp_1_7 |

**SI Table 3.** PSSMs of protein domains from CDD and Pfam (Finn et al. 2016; Marchler-Bauer et al. 2017) used to classify potential TEs identified by CARP. If a CARP consensus sequence contained a domain from each of the groups associated with that TE class/subclass it was initially classified as belonging to it. As domains present in ERVs and LTR retrotransposons overlap, and because there are many subclasses of LINE, CENSOR was used to further classify elements.

SI **Dataset 1.** Annotation of HT LINEs outputusing CENSOR 4.2.29 [(Kohany et al. 2006)](https://www.zotero.org/google-docs/?cC6Cbt) in combination with RepBase [(Bao et al. 2015)](https://www.zotero.org/google-docs/?BK6Rx3).

SI **Dataset 2. Megablast** 2.7.1 [(Altschul et al. 1990; Camacho et al. 2009)](https://www.zotero.org/google-docs/?MbReNh) hits for all 7 repeats in 11 sea snake transcriptomes from Crowe-Riddell, 2019. Results below 250bp in length and <95% identity discarded.

SI **Dataset 3.** Latin species names and versions of all public genomes used. All were downloaded from GenBank (Benson et al., 2017).

SI **Dataset 4.** Number of repeats similar to Rex1-Snek_1H1, Rex1-Snek_1H2, Rex1-Snek_1H3, Rex1-Snek_2, RTE-Snek_1, RTE-Snek_2, RTE-Kret and Proto2-Snek found in all species search using BLASTN+ 2.7.1 [(Altschul et al. 1990; Camacho et al. 2009)](https://www.zotero.org/google-docs/?7nBIZA) with relaxed parameters (see Methods).

**SI Dataset 5.** Sequences of the 7 horizontally transferred LINEs found in *Aipysurus laevis* and the one horizontally transferred LINE found in *Laticauda colubrina.*

*SI* **Dataset 6.** Sequences of all manually curated Rex1s, RTEs and Proto2s from all species.

**SI Dataset 7.** Rex1 newick tree. Phylogeny created using RAxML [(Stamatakis 2014)](https://www.zotero.org/google-docs/?FvH7rl) from a multiple sequence alignment of the four Rex1s found in *Aipysurus laevis*, all manually curated Rex1s and all Rex1s from RepBase generated using MAFFT [(Katoh and Standley 2013)](https://www.zotero.org/google-docs/?vHUX4i) and trimmed using Gblocks [(Talavera and Castresana 2007)](https://www.zotero.org/google-docs/?hFhEiC).

**SI** **Dataset 8.** Newick tree of all RTE-like LINEs. _1, RTE-Snek_2Phylogeny created using RAxML [(Stamatakis 2014)](https://www.zotero.org/google-docs/?bGFi8O) from a multiple sequence alignment of Proto2-Snek, RTE-Snek_1, RTE-Snek_2, RTE-Kret, all manually curated Rex1s and all Rex1s from RepBase. generated using MAFFT [(Katoh and Standley 2013)](https://www.zotero.org/google-docs/?h1h3Rg) and trimmed using Gblocks [(Talavera and Castresana 2007)](https://www.zotero.org/google-docs/?Bkkw1c)

Dataset S9. A list of the species TimeTree was unable to resolve, and species it used as substitutes nodes for those queried but absent from the TimeTree database.

**SI References**

[Altschul SF, Gish W, Miller W, Myers EW, Lipman DJ. 1990. Basic local alignment search tool. *J. Mol. Biol.* 215:403–410.](https://www.zotero.org/google-docs/?e37IQt)

[Bao W, Kojima KK, Kohany O. 2015. Repbase Update, a database of repetitive elements in eukaryotic genomes. *Mob. DNA* 6:11.](https://www.zotero.org/google-docs/?e37IQt)

Benson DA, Cavanaugh M, Clark K, Karsch-Mizrachi I, Lipman DJ, Ostell J, Sayers EW. 2013. GenBank. Nucleic Acids Res 41:D36–D42.

[Camacho C, Coulouris G, Avagyan V, Ma N, Papadopoulos J, Bealer K, Madden TL. 2009. BLAST+: architecture and applications. *BMC Bioinformatics* 10:421.](https://www.zotero.org/google-docs/?e37IQt)

[El-Gebali S, Mistry J, Bateman A, Eddy SR, Luciani A, Potter SC, Qureshi M, Richardson LJ, Salazar GA, Smart A, et al. 2019. The Pfam protein families database in 2019. *Nucleic Acids Res.* 47:D427–D432.](https://www.zotero.org/google-docs/?e37IQt)

Finn RD, Coggill P, Eberhardt RY, Eddy SR, Mistry J, Mitchell AL, Potter SC, Punta M, Qureshi M, Sangrador-Vegas A, et al. 2016. The Pfam protein families database: towards a more sustainable future. Nucleic Acids Research 44:D279–D285.

[Gruber M, Söding J, Lupas AN. 2006. Comparative analysis of coiled-coil prediction methods. *J. Struct. Biol.* 155:140–145.](https://www.zotero.org/google-docs/?e37IQt)

[Katoh K, Standley DM. 2013. MAFFT Multiple Sequence Alignment Software Version 7: Improvements in Performance and Usability. *Mol. Biol. Evol.* 30:772–780.](https://www.zotero.org/google-docs/?e37IQt)

[Kohany O, Gentles AJ, Hankus L, Jurka J. 2006. Annotation, submission and screening of repetitive elements in Repbase: RepbaseSubmitter and Censor. *BMC Bioinformatics* 7:474.](https://www.zotero.org/google-docs/?e37IQt)

[Lawrence M, Huber W, Pagès H, Aboyoun P, Carlson M, Gentleman R, Morgan MT, Carey VJ. 2013. Software for Computing and Annotating Genomic Ranges. *PLoS Comput. Biol.* [Internet] 9. Available from: https://www.ncbi.nlm.nih.gov/pmc/articles/PMC3738458/](https://www.zotero.org/google-docs/?e37IQt)

[Lee S, Cook D, Lawrence M. 2019. plyranges: a grammar of genomic data transformation. *Genome Biol.* 20:4.](https://www.zotero.org/google-docs/?e37IQt)

[Marchler-Bauer A, Bo Y, Han L, He J, Lanczycki CJ, Lu S, Chitsaz F, Derbyshire MK, Geer RC, Gonzales NR, et al. 2017. CDD/SPARCLE: functional classification of proteins via subfamily domain architectures. *Nucleic Acids Res.* 45:D200–D203.](https://www.zotero.org/google-docs/?e37IQt)

[Marchler-Bauer A, Bryant SH. 2004. CD-Search: protein domain annotations on the fly. *Nucleic Acids Res.* 32:W327–W331.](https://www.zotero.org/google-docs/?e37IQt)

[RStudio Team. 2015. RStudio: integrated development for R. RStudio, Inc., Boston, MA Available from: http://www. rstudio. com](https://www.zotero.org/google-docs/?e37IQt)

[Stamatakis A. 2014. RAxML version 8: a tool for phylogenetic analysis and post-analysis of large phylogenies. *Bioinformatics* 30:1312–1313.](https://www.zotero.org/google-docs/?e37IQt)

[Talavera G, Castresana J. 2007. Improvement of Phylogenies after Removing Divergent and Ambiguously Aligned Blocks from Protein Sequence Alignments. *Syst. Biol.* 56:564–577.](https://www.zotero.org/google-docs/?e37IQt)

[Wickham H. 2011. ggplot2. *Wiley Interdiscip. Rev. Comput. Stat.* 3:180–185.](https://www.zotero.org/google-docs/?e37IQt)

[Zimmermann L, Stephens A, Nam S-Z, Rau D, Kübler J, Lozajic M, Gabler F, Söding J, Lupas AN, Alva V. 2018. A Completely Reimplemented MPI Bioinformatics Toolkit with a New HHpred Server at its Core. *J. Mol. Biol.* 430:2237–2243.](https://www.zotero.org/google-docs/?e37IQt)
